# Key Parameters for the Rational Design, Synthesis, and Functionalization of Biocompatible Mesoporous Silica Nanoparticles

**DOI:** 10.3390/pharmaceutics14122703

**Published:** 2022-12-02

**Authors:** Marta Florensa, Marina Llenas, Esperanza Medina-Gutiérrez, Stefania Sandoval, Gerard Tobías-Rossell

**Affiliations:** Institut de Ciència de Materials de Barcelona (ICMAB-CSIC), Campus UAB, 08193 Barcelona, Spain

**Keywords:** drug delivery, biomedical applications, sol-gel, biomedical imaging, vaccines, theragnostic agents, cancer, MSNs

## Abstract

Over the last few years, research on silica nanoparticles has rapidly increased. Particularly on mesoporous silica nanoparticles (MSNs), as nanocarriers for the treatment of various diseases because of their physicochemical properties and biocompatibility. The use of MSNs combined with therapeutic agents can provide better encapsulation and effective delivery. MSNs as nanocarriers might also be a promising tool to lower the therapeutic dosage levels and thereby to reduce undesired side effects. Researchers have explored several routes to conjugate both imaging and therapeutic agents onto MSNs, thus expanding their potential as theranostic platforms, in order to allow for the early diagnosis and treatment of diseases. This review introduces a general overview of recent advances in the field of silica nanoparticles. In particular, the review tackles the fundamental aspects of silicate materials, including a historical presentation to new silicates and then focusing on the key parameters that govern the tailored synthesis of functional MSNs. Finally, the biomedical applications of MSNs are briefly revised, along with their biocompatibility, biodistribution and degradation. This review aims to provide the reader with the tools for a rational design of biocompatible MSNs for their application in the biomedical field. Particular attention is paid to the role that the synthesis conditions have on the physicochemical properties of the resulting MSNs, which, in turn, will determine their pharmacological behavior. Several recent examples are highlighted to stress the potential that MSNs hold as drug delivery systems, for biomedical imaging, as vaccine adjuvants and as theragnostic agents.

## 1. Introduction

Nanoscience and nanotechnology have developed into the backbone of modern research in last years as a result of the introduction of new and improved technologies [1]. Ranging from basic to applied sciences, with a focus on nanoengineering new materials, [2] nanotechnology encompasses fundamental research, engineering and technology and entails the modeling, construction, characterization and manipulation of matters’ properties at the nano-scale.

Nanomaterials are typically identified as materials with at least one dimension in the range of 1–100 nm [3]. Their physical, chemical and biological properties might be completely different from those of bulk matter [4]. The unique features of these nanostructures are attributed to quantum confinement or surface effects [5], which result in interesting and potentially useful catalytic, magnetic, optical and electrical properties [6]. Consequently, nanomaterials have long been a subject of interest worldwide [7], allowing for the development of a diverse range of advanced materials that brings together the demands of high-tech and scientific applications [4], which are already revolutionizing industries such as electronics, medicine and consumer products [8].

**Figure 1:**
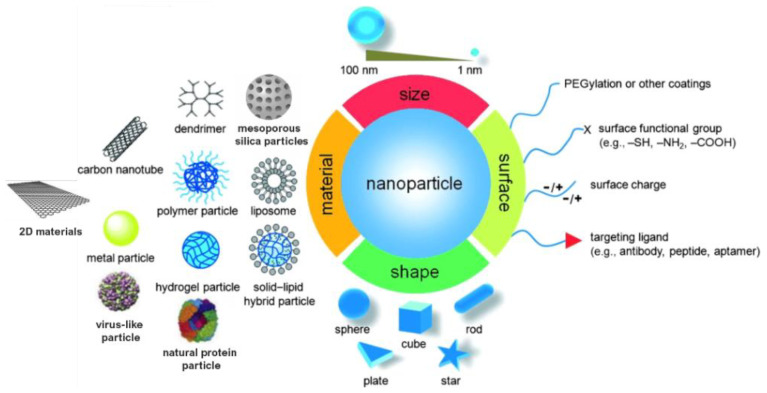
Material, shape, size and surface properties play an important role on the biophysicochemical properties of nanoparticles. Adapted from [10,11]. Copyright © 2022 WILEY-VCH Verlag GmbH and Co. KGaA, Weinheim.

Monitoring the size and shape of nanoparticles can help to improve their performance in specific applications (Figure 1). Similarly, characteristics such as chemical composition, charge and the surface can be altered to suit specific purposes. Additionally, functional groups, hydrophilicity, attachment and conjugation with targeting ligands also comprise the essential parameters that need to be considered [9,10].

Nanomaterials can be broadly classified according to their organic or inorganic composition. Organic nanomaterials include polymeric nanostructures, dendrimers, lipid bilayers and virus and proteic particles, whereas inorganic nanomaterials are composed of metals, oxides, nitrides, halides, carbonates and chalcogenides, to name some [12]. Among the inorganic materials, nanosized silica has been extensively studied and employed in a wide range of applications [13].

Silicon is widely distributed throughout the world, accounting for *ca.* 40% of all known minerals. It occurs naturally in a variety of forms in the environment, most frequently in combination with oxygen (both amorphous and crystalline) or hydroxides. Silicon is also found dissolved in the oceans in the form of silicic acid and in living organisms, such as sponges, greases and algae (diatoms) [14]. Silica also comprises one of the most complex and abundant families of materials, occurring naturally in a variety of minerals (including quartz, flint, opal and silicates) and as synthetic products (fused-quartz, fumed-silica, silica-gel, etc.). The Si atom exhibits tetrahedral coordination within the majority of silicates, consisting of four oxygen atoms surrounding a central Si atom [8]. Amorphous silica is primarily prepared through simple manufacturing procedures on a large scale and its properties can be tailored to a wide range of applications. Silica combines a high degree of chemical stability, low toxicity and high biocompatibility with a plethora of possibilities for modifying its physical and chemical properties, as well as the possibility of further modification via functional group coupling, binding of biomolecules or dye doping [15]. Therefore, monodispersed colloidal silica particles with uniform shape, size and composition have demonstrated significant potential, not only in physical chemistry, being used to investigate the dynamic behavior and stability of particulate systems, but also in industries, including pigments, photographic emulsions, ceramics, catalysts, etc. [16].

Porous silica presents a customizable mesoporous structure and pore volume, as well as a high specific surface area. These properties enable the encapsulation of a variety of compounds, including gas and heavy metal ion adsorbents, bioimaging probes and therapeutic agents, such as drugs [17]. In this respect, ordered mesoporous materials, such as the M41S family, comprise an emerging and significant class of inorganic oxides with enormous potential for molecular sieving, catalysis and adsorption processes [6].

Numerous researchers have described various synthetic methodologies for the preparation of mesoporous oxide materials, proposing various mechanisms for the formation of their porous structures. Different factors influence the porous structure formation and affect the size, distribution, and ordering of pores, including precursors (alkoxides, metal salts, etc.), surfactants as structure-directing agents and reaction parameters (pH, temperature, etc.). Their size and shape, which can, for instance, take the form of spheres, rods, or wormlike structures, can also be controlled by adjusting the molar ratio of silica precursors and surfactants, the pH, adding co-solvents or organic swelling agents and introducing alkoxysilane precursors [16,18]. Notably, the fabrication of mesoporous silica nanoparticles is straightforward, scalable, economic and controllable.

This review is intended to provide an overview of the synthesis, formation mechanisms, characterization, modification and applications of silica nanoparticles, in order to highlight the recent trends on silicates that are of high significance. Special emphasis is paid to the relationship between the parameters employed for their synthesis and the properties of the resulting particles, both physicochemical and biological. To begin, the routes used to produce MSNs, as well as the surface functionalization methods employed to modify their surface and composition, are discussed. Next, a brief overview of the applications of MSNs is provided, focusing on their use as drug delivery systems (DDS) and bioimaging, the combination of which allows for their use as theranostic agents. Finally, a discussion of the most significant physiochemical properties of MSNs that affect their in vivo fate is presented, followed by a description of their potential biological applications using various examples.

## 2. Silica Nanoparticles

The use of SiO_2_ nanoparticles (NPs) has increased in recent years, due to the diversification of their properties, thus widening their potential applications. The ease of preparation and surface modification confer the material versatility, being useful in a variety of fields, such as catalysis, semiconductors, pigments, pharmacy, electronics, detergents, cosmetics and sensors. SiO_2_ NPs can be grown to lead different nanostructures, namely solid nanospheres and mesoporous and hollow silica nanoparticles (HSNs), also adopting diverse morphologies (cubic systems, nanorods, etc.); the characteristics of the resulting particles being closely linked to the synthetic approach.

### 2.1. Strategies for the Preparation of Silica NPs

Two main approaches, the *viz.* top-down and bottom-up techniques, have been proposed to obtain silica nanoparticles. In the first one, the dimension of the original size is reduced by breaking down the constituents of bulk materials. Conversely, the bottom-up method is commonly used to produce silica nanoparticles from the atomic or molecular level to the nano-/micro-scale [4]. Due to the advantages of the bottom-up approach, which includes the ability to control the particle size and morphology, this is the strategy most widely employed for the synthesis of inorganic nanostructures, including silica NPs. This protocol also leads to a narrower size distribution through controlling the reaction parameters [2].

#### 2.1.1. The Sol-Gel Method

The sol-gel approach entails the hydrolysis, followed by the condensation of alkoxide monomers into a colloidal solution (sol), acting as the precursor to form an ordered network (gel) of polymer or discrete particles [1,19,20].

In 1956, Kolbe [21] described the formation of silica particles by reacting tetraethyl silicate in an alcoholic solution containing water in the presence of certain bases. It was observed that, using very pure reagents, the reaction, which proceeds slowly, leads to the formation of uniform spherical silica nanoparticles. Later, Stöber et al. [22] developed a system that allowed the controlled growth of spherical silica particles with uniform sizes (50 nm–2 μm) via the hydrolysis of alkyl silicates and their subsequent condensation in low molecular-weight alcohols as the solvent, while using ammonia as the morphological catalyst.

Generally, the formation of silica particles can be divided into two steps, namely nucleation and growth. To describe the growth mechanism of silica, two models have been proposed. The monomer addition model predicts that the particle growth occurs through the addition of other hydrolyzed monomers after an initial nucleation state. The aggregation model describes that primary particles (nuclei) aggregate together to form dimers, trimers and progressively larger particles (secondary particles) [4]. At the same time, the nature of the catalyst strongly influences both the rate and mechanism of hydrolysis and condensation. In the case of an acid-catalyzed reaction, the hydrolysis becomes faster than the condensation, resulting in the formation of numerous small silica particles. Conversely, in the base-catalyzed reaction, the condensation proceeds much faster than the hydrolysis step, leading to larger silica nanoparticles [23]. The acid-catalyzed mechanisms are preceeded by the rapid deprotonation of the substituents bonded to Si, whereas under basic conditions, the hydroxide ion serves as a nucleophile that attacks the silicon atom center of the tetraalkoxysilane. The result of this step is a silanol and an alkoxide ion [12,24].

**Scheme 1:**
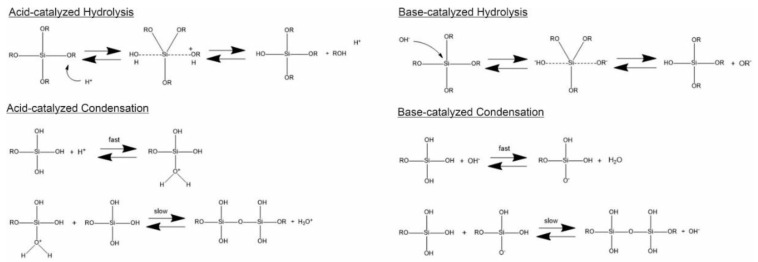
Hydrolysis and condensation in acidic and basic conditions. Adapted from [26]. Copyright © 2022, American Chemical Society.

In particular, the first step of hydrolysis induced by the acid catalyst is the electrophilic attack of the proton on an oxygen atom of the silica precursor, leading to a positive charge on it. This attack makes the bond between the silicon and the attacked oxygen (Si-O) more polarized and facilitates its cleavage, thus producing an alcohol as the leaving group. Therefore, an increase in the water-to-alkoxide ratio increases the rate of hydrolysis [25]. Following the hydrolysis reaction, the condensation reaction occurs immediately [14]. During the condensation, the proton electrophilically attacks the oxygen of the silanol group. This phenomenon causes the oxygen of the silanol to become positively charged and a siloxane bridge is formed because of condensation between both protonated and unprotonated silanol groups [25]. On the other hand, the base-catalyzed hydrolysis proceeds via a nucleophilic attack of the hydroxyl group on the silicon atom of the alkoxide, while the subsequent release of an OR^−^ as the leaving group is facilitated. On condensation, the hydroxyl group of intermediate [Si (OC_2_H_4_)_4−x_ (OH)_x_] reacts with either the ethoxy group of other alkoxysilanes (alcohol condensation) or the hydroxyl group of another hydrolysis intermediate (water condensation) to form Si-O-Si bridges (Scheme 1) [14].

**Figure 2:**
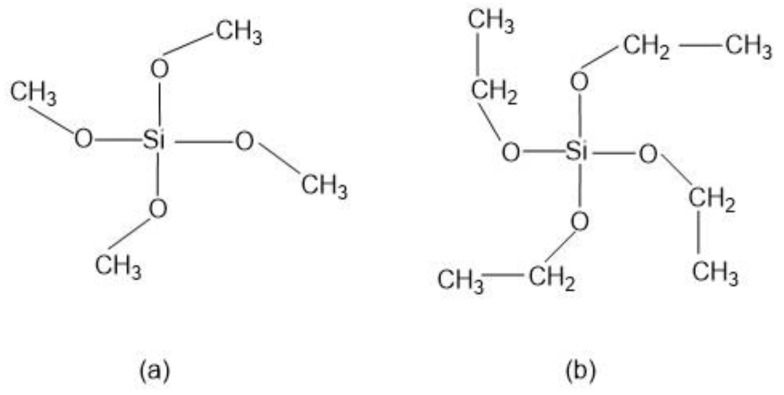
Alkoxysilanes such as (**a**) tetramethoxysilane (TMOS) and (**b**) tetraethoxysilane (TEOS) are extensively used to produce silica gels.

Numerous alkoxysilane precursors (Si(OR)_4_) have been reported, including tetramethyl orthosilicate (TMOS) or tetraethyl orthosilicate (TEOS) (Figure 2), which undergo hydrolysis and condensation reactions upon specific pH conditions [27].

The hydrolysis step leads to the generation of silanol groups (Si-OH), the mechanism being mainly conditioned by the catalyst, while its rate depends on the pH, the solvent used and the ratio of water-to-alkoxide. Since alkoxysilanes are not soluble in water, organic co-solvents are required to facilitate hydrolysis. In the condensation step, the silanol group interacts with an alkoxide or silanol group to form a strong siloxane bond (Si-O-Si), thus resulting in the loss of one molecule of alcohol (ROH) or one molecule of water [25].

Silica particles are also synthesized via the sol-gel method using water-in-oil (W/O) emulsions. Reverse micelles in oil reflect the size, shape and size distribution of the particles, as they act as templates for their growth [28]. In the case of reverse microemulsions, the surfactant molecules are dissolved in organic solvents to form spherical micelles. In the presence of water, the polar head groups adopt the lowest energy position and organize to form water-containing microcavities. During the synthesis, silica nanoparticles can be grown inside the reverse micelles by controlling the addition of silicon alkoxides and the catalyst into the medium. The diffusion of the alkoxide into water droplets outcome in hydrolysis of the alkoxide and the formation of alcohol and oxy-hydroxy-silicate species [29]. The main drawbacks of the reverse microemulsion approach are the high costs and the complications associated with the removal of surfactants in the final products [4].

#### 2.1.2. Other Methods

Until now, the sol-gel method has been the most straightforward and effective method for synthesizing monodispersed silica spheres. However, over the years, numerous research groups have introduced improvements in the hydrolysis method to achieve uniform shape and monodisperse distribution [16] and novel routes have been explored for the preparation of silica nanoparticles that have short reaction times, excellent reproducibility and are simple to prepare [30]. Alternative processes worth mentioning include microwave, ultrasound and evaporation-induced approaches, self-assembly (EISA), hydrothermal and solvothermal, or oxidative processes, which have also been explored to optimize the production of pure silica particles [31]. Despite the fact that a detailed explanation of each methodology is outside the scope of the present review, we believe that energy-assisted syntheses present some unique benefits.

Energy-assisted approaches, namely the microwave- or ultrasound-induced formation of SiO_2_ NPs represent attractive, although not generalized, techniques to obtain functional nanostructures. Due to the characteristics of the incident energy source (300 MHz–300 GHz), microwave-assisted synthesis requires almost negligible working times and yields high quality and reproducible NPs (in terms of size distribution and shape). The formation process occurs due to the alignment with the electromagnetic field of the polar and/or charged molecules present within the irradiated system, which, as an effect of the energy, increases its temperature (due to the vigorous agitation of the species). The temperature gradient profile generated during the process is highly homogeneous, favoring aspects as a high reaction yield by employing much shorter times than sol-gel methods or the formation of species with narrow size distributions. The possibility of tuning the characteristics of the incident energy also allows controlling the conditions of the system, thus improving the synthetic approach, such as for instance, reducing the times of the reaction or enhancing the selectivity towards a selected material, therefore minimizing the need of further purification steps for the removal of undesired products. Different Si precursors have been employed to obtain SiO_2_ nanospheres using microwave, for instance, TMOS in the presence of HCl as a catalyst. Noticeably, the approach requires employing a solvent, such as acetone, which allows the absorption of the incident radiation by the reactants. Thus, the SiO_2_ NPs were obtained using times as short as 1 min and mild conditions (125 °C). The size of the resulting NPs depends on the concentration, time of treatment and irradiation power [32]. Despite the fact that the protocol can be applied to a wide variety of nanostructures, the main drawbacks are related to the low scalability of the approach or the poor homogeneity of the surface of the obtained NPs [24]. On the other hand, taking advantage of the efficiency of the energy-assisted methodologies, a hybrid approach, consisting on the combination of the well-known sol-gel protocol and ultrasound allowed controlling the size of the core NPs (d = 13 nm), thus confirming the versatility of the technique and the possibility of controlling the morphological characteristics of the products [33].

### 2.2. Mesoporous Silica Nanoparticles

According to the IUPAC definition, porous materials can be classified into three groups based on their pore sizes, namely microporous, mesoporous and macroporous ones, with corresponding pore diameters of <2 nm, 2–50 nm and >50 nm, respectively [34].

In 1990, Yanagisawa et al. [35] reported the condensation of silicate layers to form a structure of 3D “nanoscale pores” [36]. The protocol, based on the intercalation of alkyltrimethylammonium cations into kanemite silicate, followed by calcination (removal of organic species), led to the development of the characteristic MCM-41 mesoporous materials. On their basis, in 1992, Mobil Corporation Laboratories designated a new family of amorphous and ordered mesoporous inorganic materials with exceptionally large and uniform pore conformations, narrow pore size distributions and large surface areas: the M41S (molecular 41 sieves) [34,36,37]. This family includes two main groups: those with a three-dimensional interconnected porous structure (cubic-ordered pore structure) designated as MCM-48 and those with a one-dimensional and hexagonally ordered pore structure, known as MCM-41 and MCM-50, which have an unstable lamellar structure [19,38]. Following their discovery, significant efforts have been made to control the properties (particularly, the pore size and morphology) of mesoporous silica. Through this research, in 1995, new families of mesoporous silica systems (MSS), namely SBA, MSU and FSM, were developed [39]. Later, in 1998, the search for a mesoporous material with a hexagonal array of pores resulted in the formation of the Santa Barbara Amorphous No.15 (SBA-15), a research gambit in mesoporous material development [20]. SBA-15 demonstrated large pore sizes (up to 30 nm), as well as thermal, mechanical and chemical resistance, establishing it as the preferred mesoporous material for catalysis’ applications [25].

**Figure 3:**
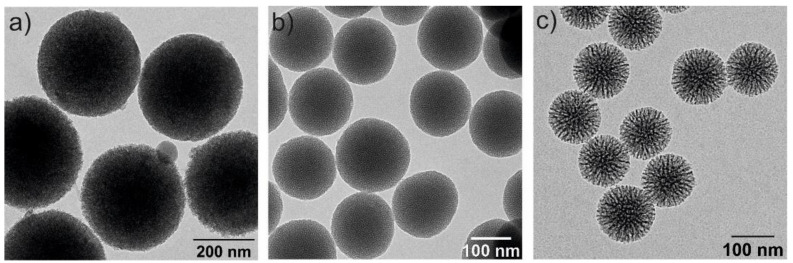
Transmission electron microscopy (TEM) images of three spherical MSNs with different particle and pore sizes: (**a**) 350 nm and 2–3 nm of pore diameter, (**b**) 150 nm size and 2–3 nm pore diameter and (**c**) 100 nm and 6–8 nm pore diameters.

Among the mesoporous silica systems, MSNs offer outstanding advantages over non-porous silica nanoparticles, such as large pore volume (0.6–1.5 cm^3^/g), high surface area (700–1000 m^2^/g), tunable particle sizes, tailorable pore diameter (2–10 nm), high thermal, chemical and biological stabilities, ease of functionalization, biodegradability and good biocompatibility [40,41]. MSNs have a high rate of mass transport, a high affinity for substrates and a high dispersibility in solutions [42]. Additionally, they have two functional surfaces, the inner one, which corresponds to the pore channels and the external surface, which is highly susceptible to be selectively functionalized [43]. The particle size, pore diameter and characteristics of the silica NPs depend on the synthesis conditions [44]. Some examples of silica NPs, analyzed by transmission electron microscopy, are illustrated in Figure 3.

Silica spheres possessing nanopores of well-defined size and connectivity are of significant interest, for instance, for the use in catalysis, chromatography, for the controlled release of drugs and as hosts for optically active compounds [45].

#### 2.2.1. Synthesis of Mesoporous Silica NPs

Since their development, a variety of mechanisms have been proposed to explain the formation of MSNs [2]. At the most fundamental level, all of these models imply that supramolecular assemblies of surfactants serve as templates for the formation of porous structures in the inorganic matrix [19].

Grun et al. reported the synthesis of MSNs with multiple dimensions, pore sizes, structures and morphologies using a modified Stöber’s method [22,25]. Constant research has resulted in variations of the synthesis conditions and methods to yield stable and monodisperse MSNs [1]. Based on the previously detailed methodologies, mesoporous silica spheres can be synthetized by introducing templating surfactants, such as cetyltrimethylammonium bromide (CTAB).

**Figure 4:**
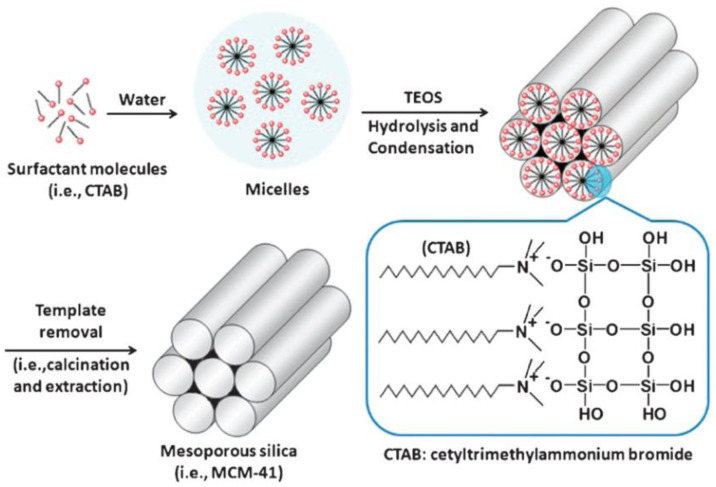
Schematic representation of the mechanism of formation of MCM-41 using cetyltrimethylammonium bromide (CTAB) as cationic surfactant [48]. Copyright © 2022, Royal Society of Chemistry.

Surfactant molecules are highly active components that progressively create more complex structures as concentration increases, while seeking a state of equilibrium or minimum energy [23]. At low concentrations, the surfactants exist as single molecules. By increasing their concentration in aqueous solutions, these molecules combine to form micelles to reduce the contact between the hydrophobic hydrocarbon chains and water, thus reducing the surface tension. In general, a silicon alkoxide promotes the formation of the silica structure outside the micelles and the presence of a catalyst facilitate the hydrolysis and condensation of silica precursors to form a network of siloxane bonds [46,47]. As an example, Figure 4 illustrates the formation of MCM-41. Initially, under a certain concentration, the surfactant molecules are free in the medium. Once their concentration increases and they reach the critical micelle concentration (CMC), the surfactants aggregate to form an isotropic micelle system. The concentration process continues and hexagonal assemblies appear close together, resulting in the formation of the hexagonal phases. The coalescence of adjacent, mutually parallel cylinders produces the lamellar phase. The formation of a particular phase depends not only on concentrations, but also on pH, temperature, ionic strength, solvent, the presence of other compounds, the nature of the surfactant itself (including the length of the hydrocarbon chain), the hydrophilic head group and the counter ion, in the case of ionic surfactants [25]. Finally, in order to incorporate payloads into the pores, the surfactant channels might be removed by calcination or via chemical treatment.

##### Sol-Gel Process through Template-Assisted Technique

The template-assisted technique is the most popular formation mechanism for MCM-41, first postulated in 1992 by the Mobil scientists. According to this mechanism, surfactant molecules, such as alkyltrimethylammonium salts, form hexagonal arrays of micellar rods, which play a templating role (structure-directing agent). Afterwards, the silicate species assemble in between the surfactant tubules through the sol-gel process to form the inorganic framework [21,48,49]. Accordingly, the final product is a silicate skeleton that contains voids and mimics these mesophases [36]. Additionally, silica/surfactant mesophases are formed in the solution by one of two complementary approaches: (1) the cooperative assembly of small silicate species with micelles and individual surfactant molecules and (2) liquid crystal templating (LCT) of molecular inorganic species around a preformed, spatially extended organic superstructure [50,51]. The silicate species added to the reaction mixture may influence the ordering of the isotropic rod-like micelles to form the desired liquid crystal phase (hexagonal mesophase) [36]. On the other hand, the surfactant creates a hexagonal structure before the silica precipitates around this template, forming the mesoporous structure [20].

In fact, hexagonal ordering may be mediated by the interaction of silicate species with surfactants and both, the formation of surfactant liquid crystals and the assembly of silicate ions may occur simultaneously. It has been hypothesized that, during the formation of cylindrical surfactant/silica aggregates, silicate ions enter the particle by migrating from the surface toward the center of the particles (by a controlled diffusion process). Meanwhile, the surfactant molecules must enter through the cylinders of the aggregates, resulting in a continuous increase in organic content and channel diameter. This occurs more readily near the particle surface, where the network tension is lower than in the center [48,49].

Frasch and co-workers [52] proposed a similar model to the one reported by Cai et al. [53], in which silicate/surfactants rod-micelles form in the first step and then pack together in an ordered fashion to form the mesoporous nanoparticles [54].

Once the MSN structure has been obtained, removal of the surfactant or organic material is essential. This is usually achieved by calcination or solvent extraction. In the case of calcination, the synthesized MSNs are heated to temperatures between 300 °C and 600 °C [23,53]. However, at high temperatures, the particles can undergo surface modifications. Specifically, the Si-OH bonds on the surface of the MSNs react to form siloxane bonds. This constricts the surface and pores, modifying the pore size and making the particle more hydrophobic. Solvent extraction can also be used but the conditions will depend on the surfactant nature and whether the reaction occurred under acidic or basic conditions [23]. For the synthesis of mesoporous materials by sol-gel processes, different templates, such as cation surfactants, triblock copolymers and small organic molecules can be used as structure-directing agents [20].

Beside the sol-gel/template assisted synthesis of MSNs, the most widely investigated approaches are the microwave technology (which saves both energy and time, and the operation of the system is relatively simple) [55], hydrothermal and EISA. In all cases, it is necessary to introduce a templating agent to allow the formation of a nanoporous structure, in which the characteristics of the cavities (size, shape, orientation, etc.) are related to the configuration of the organic mold. 

#### 2.2.2. Factors Influencing the Size and Shape of Mesoporous Materials

The morphological and physicochemical properties of MSNs can be controlled by varying the synthesis conditions. MSNs are primarily formed in an ordered fashion from surfactants under acidic, basic or neutral conditions, which dictates their shape and size [48]. MSNs can be synthesized using anionic, cationic, neutral surfactants or non-surfactant template pathways [56]. The diameter of the pores can be controlled by changing the length of the template molecule. Size, shape and porosity can be tuned by changing the silica source (e.g., colloidal silica or TEOS), surfactants (e.g., hexadecylamine (had) and cetyltrimethylammonium bromide (CTAB)), auxiliary compounds, or reaction conditions (e.g., solvent, temperature, time, stirring speed, reactant mole ratio and pH of the medium), thus affecting, at the same time, the properties and stability of the material [1,25].

**Figure 5:**
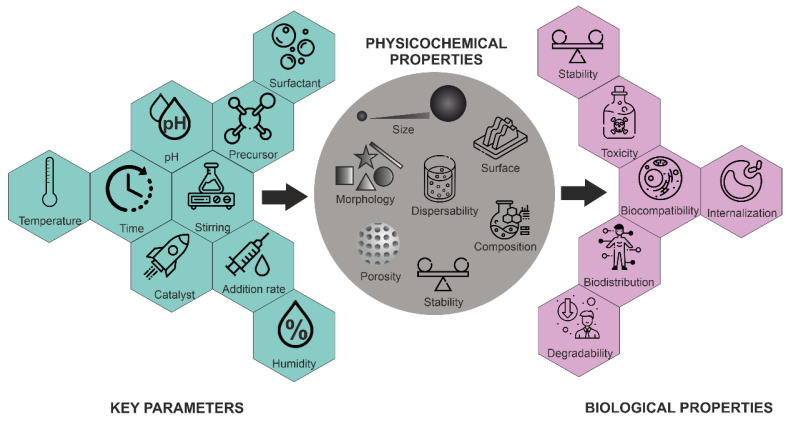
Influence of key parameters on the physico-chemical and biological properties of MSNs.

Morphology control is extremely important for several applications. Not only have a variety of spherical or quasi-spherical MSNs with various porous and bulk properties been reported [45], but different morphologies, including thin films, hexagonal prisms, spirals, dodecahedrons and hollow tubular shapes of mesoporous silica, have also been synthesized. The main parameters for the synthesis of MSNs and their effects on particle properties are summarized in Figure 5.

##### pH

The hydrolysis and condensation rates of silica species are strongly pH-dependent, which dictates both the reaction’s kinetics and the final network structure [25]. Therefore, pH is an important parameter and plays a critical role. It influences the synthetic chemistry of mesoporous materials, particularly affecting the charge of inorganic precursor species and surfactant head groups and, consequently, their mutual interaction [19]. At high pH values, the negatively charged inorganic precursors interact preferentially with the positively charged groups of the surfactants, resulting in condensation on the solid organic-inorganic mesoporous structures. In general, in a strongly acidic medium, the rate of the hydrolysis of TEOS is faster than the condensation. By changing the pH, a phase transformation of silica from the lamellar to the hexagonal phase has been reported [19]. The formation mechanism of mesophases under acidic and basic conditions is completely different because negatively charged silicate ions act as counter ions above the isoelectric point (pH > pI~2.0), while below the isoelectric point, positively charged silicate ions act as counter ions [19,26]. In addition, the dissociation constant (pK_a_) of the silanol groups (Si-OH)—which are present at the silica surface—has been determined in the range 4–5.5, indicating the dissociation of Si-OH groups, forming negatively charged Si-O^−^ groups at the surface of silica [57].

**Figure 6:**
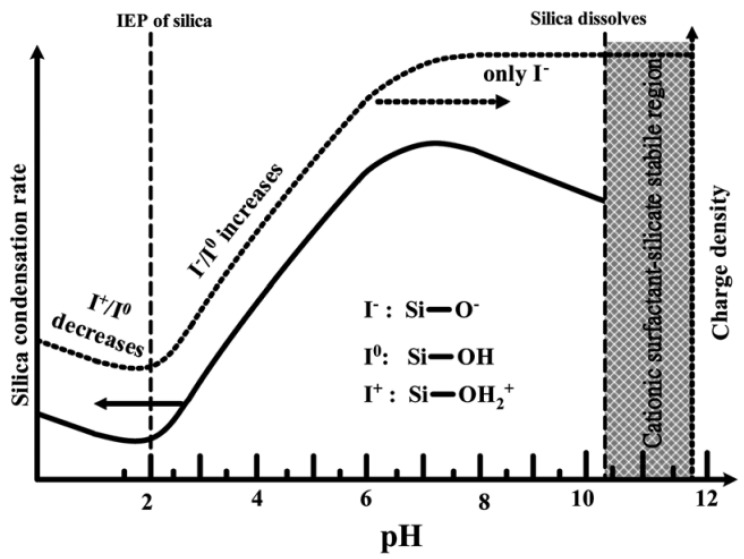
Effect of pH on the silica condensation rate and silica surface charge properties [46]. Copyright © 2022, Royal Society of Chemistry.

As shown in Figure 6, at pH values lower than the isoelectric point (pH ~2), the condensation is acid-catalyzed [58], the silica species are also positively charged and the charge density increases as the pH decreases [46]. At pH > 2, the condensation rate increases with a pH up to about 7.5 [58]. At this point, the silica species become negative and the charge density of silicates increases along with increasing pH, due to the favored nucleophilic attack. At pH ~2–7, silicates with a negative charge density tend to assemble with positively charged surfactants or neutral polymers through hydrogen bonds and electrostatic interactions. Under alkaline conditions (pH >7.5), silicates with a high negative charge density can only assemble with cationic surfactants by a single, but strong, electrostatic interaction. From this point on, the condensation rate reaches a maximum and decreases for pH >7.5, due to the gradual instability of the silicates at higher pH [22,59]. Thus, the condensation reaction of silicates can be represented as follows:

–Si–O^−^ + HO–Si– → –Si–O–Si– + OH^−^ (1)

The effect of pH on the morphology of MSNs was studied by Yang et al. [60]. They demonstrated that, under mildly acidic conditions, 1–10 μm spherical mesoporous particles are formed. Furthermore, Voegtlin et al. [61] reported the room temperature synthesis of MSNs at pH values ranging from 8.5 to 12. Finally, Chiang et al. [62] studied all the parameters that can influence the particle size of MSNs, concluding that the pH played a key role [63].

##### Surfactant

Surfactant chemistry is critical for understanding the mesoporous structure formation. The pore size and structure, pore walls, or phase and symmetry of the materials are governed by the nature of the surfactant. Surfactant molecules have an amphiphilic nature, as they have a hydrophilic polar head group and a hydrophobic nonpolar hydrocarbon tail, being an active agent in an aqueous solution and showing high affinity to the surfaces and interfaces. Surfactants can be classified as anionic, cationic, zwitterionic and nonionic, according to the nature of their polar head group [19]. A homogenous spherical particle size distribution can be achieved at low concentration of CTAB (as cationic surfactant). Cai et al. [53] generated MSNs with different shapes, such as spheres, rods and micrometer-sized oblate silica by tuning the concentration of TEOS, NaOH/NH_4_OH and CTAB [1].

The pore width can also be adjusted, either during the synthesis or by post-synthesis hydrothermal treatments [64]. The pore size is mainly determined by the hydrophobic chain length of the surfactant. Different methods have been described to control pore size by the addition of organic auxiliary molecules or by using swelling agents, such as mesitylene, which solubilizes in the hydrophobic region, increasing the micellar size [59]. For instance, substituting the dodecyltrimethylammonium ion for a hexadecyltrimethylammonium ion (commonly known as CTAB) produced a sample of MCM-41 with 2–3 nm pore diameters [65]. Since additives can generate an imbalance in the reaction medium, post-synthesis hydrothermal treatment is an alternative to increase the pore size without inducing changes in the size and morphology of the preformed particles [64]. Furthermore, the use of co-solvent organic molecules, such as 1,3,5-trimethylbenzene (TMB), induces the formation of expanded pore sizes in MCM-41 [56].

Considering different modes of interaction between the silica precursors and surfactants, several models have been proposed [19]. Electrostatic charge-matching between cationic surfactants (S^+^) and anionic inorganic precursors (I^−^) is particularly effective for generating mesostructures with hexagonal, cubic or lamellar symmetry.

Schüth, Stucky and co-workers [66] included a charge-reversed S^−^ I^+^ pathway between anionic surfactants, such as sulfonates, phosphonates, carboxylates and cationic precursors. Tanev and Pinnavaia [67] demonstrated that the assembly of hexagonal mesoporous metal oxides can be achieved between neutral amine surfactants (S^0^) and neutral inorganic precursors (I^0^) [68]. Hexagonal mesoporous silica with wormhole-like framework structures and larger wall thicknesses can be prepared by hydrogen bond and self-assembly between a neutral amine surfactants (S^0^) or nonionic PEO-based surfactant (N^0^) with neutral inorganic precursors (I^0^) [69].

Another approach for synthesizing small particles is the addition of an optimal amount of Pluronic F127 to the surfactant, which results in a change in the structure and packing of the micelles, leading to smaller particle sizes. In addition, Pluronic F127 also enhances the dispersion of the silica precursor (TEOS) and coats and stabilizes the newly formed small MSNs, helping to protect the particles from agglomeration and oligomerization [70].

##### Silica Source

Mesoporous structures have been synthesized using several silica sources, such as sodium silicate, Ludox, fumed-silica, water glass or silicon alkoxides (e.g., TEOS and TMOS), with the latter being the most widely reported [19]. Di Renzo and co-workers [71] described the synthesis of spherical MCM-41 using various silica sources, showing their dominating role in the morphology of MCM-41 silicates [72]. K. Yano and Y. Fukushima [73] succeeded in preparing large MSNs from alkoxysilanes with longer alkyl chain lengths, such as tetrapropoxysilane, since their hydrolysis rates are slower than those of tetramethoxysilane and tetraethoxysilane [33,74]. Nooney et al. [54] prepared MSNs with sizes ranging from 65 to 740 nm by using different TEOS/surfactant ratios under diluted conditions [63]. When the TEOS concentration increases, both the hydrolysis rate and condensation rate become faster, which shortens the nucleation period. Thus, the total number of nuclei formed will be less and the final particle size of synthetic silica colloids will be relatively larger.

##### Other Factors

Other factors that affect the size and shape of the MSNs include the temperature, amount and strength of the catalyst, reaction time and type of solvent. Thus, their combination should be considered to control the morphology of these particles [30]. The solvent is particularly important when silicon alkoxides are employed for the MSNs growth. Since these substances are nonpolar and, thus, insoluble in water, they are typically dissolved in a solution of water and ethanol [18], with the mixing mode having a significant effect on the homogeneity of the Si source in the reaction medium and its interaction with both the ethanol and water molecules [75]. Zhang et al. [16] reported that the dilution of TEOS with ethanol can improve the diffusion of TEOS in the mixture of ethanol and ammonia and depress the aggregation or adhesion of nanoparticles. It was found that four times the volume of ethanol for diluting TEOS is enough to obtain monodispersed nanoparticles with a fine spherical shape.

Depending on the type of solvent used in the reaction, different particle sizes and uniformity can be obtained. Stöber, Fink and Bohn [22] studied the particles’ growth using different alcohols as solvents. Particles prepared in methanol solutions turned out to be the smallest, while the particle size increased when increasing the length of the alcohol chain. The particle size distribution also became broader when using longer-chain alcohols as solvents [76], while more uniform particles were obtained in 1:3 mixtures of methanol/n-propanol. Additionally, Stöber et al. found that the size of the silica particles that can be obtained in TEOS/ethanol/water mixtures varied from 50 nm to about 1 µm.

The particle size increases with the concentration of catalyst (with ammonia being the most widely employed one for this purpose), as well as with an increase of TEOS [14]. Ammonia, as a morphogenic catalyst, leads to more spherical morphologies [54]. Cai et al. [53] synthesized different particle morphologies by selecting sodium hydroxide over ammonium hydroxide.

The final particle size is also strongly influenced by the reaction medium’s ionic strength and the catalyst and water concentrations. This is consistent with a proposed mechanism in which the final particle size was strongly dependent on the (intermediate) particle’s stability [77]. Nanoparticles prepared by the Stöber method usually have a relatively large amount of silanol groups, which allows the nanoparticles to disperse stably, due to their strong surface charges. When the solute concentration reaches a critical point (nucleation concentration), each solute self-assembles and forms small nanoparticles (thermodynamically favored). After nanoparticle formation, the solutes are also used for nanoparticle growth, and the nanoparticles gradually become larger. Accordingly, to reduce particle size, it is necessary to promote nucleation, rather than growth, by modulating the concentration of silica precursors [30].

As it has been mentioned before, the size of MSNs can be modified by the addition of certain compounds. Anderson et al. [51] reported the influence of various co-solvents and water mixtures on the size of MSNs. Gu et al. [78] proposed the use of EG, as it can decrease the interaction between the silica species and surfactants, thus reducing the size of nanoparticles [42,74]. Similarly, the addition of a second templating agent, with a different affinity for silica, can lead to the formation of a layer surrounding the particles, thus limiting their growth. The use of organo-substituted trialkoxysilane also plays a role in controlling MSN morphology and size. By using a series of alkyl substituted silanes, Huh et al. [79] generated diverse morphologies, such as rods, beans, or spheres, with precisely controlled particle sizes. [64]. Schulz-Ekloff et al. [39] described a method for obtaining materials of a novel type, namely bimodal silicas, which contain both the MCM-41 mesopore systems, with pore sizes ranging from about 2 to 15 nm [42,60,80].

Temperature has a greater effect on the rate of hydrolysis than other factors, such as co-solvents. Therefore, a relatively high temperature, such as 80 °C, is essential when using tetraalkoxysilanes with slow hydrolysis rates [81]. As a consequence, the reaction temperature can change the properties of the particles, mainly affecting the particle size. For example, Narayan, Reema et al. [1] observed that, by increasing the temperature of the reaction from 30 to 70 °C, particle size was increased from *ca.* 29 nm to 113 nm. This could be due to the increase of the reaction rate, leading to polycondensation of the silica monomers and resulting in a dense silica structure and a larger size.

In summary, the parameters that control the size and morphology of MSNs include the rate of hydrolysis and condensation (pH-dependent) and the level of interaction between the assembled template and the silica precursor [63,64,82].

### 2.3. Hollow Silica Nanoparticles

Hollow spheres represent a special class of materials that are of interest, for instance, in the fields of medicine, pharmaceutics, materials science, catalysis and the paint industry. They can encapsulate a variety of products (for the controlled release of drugs, cosmetics, inks and dyes), protect light-sensitive compounds and to be employed in coatings, composites and fillers [80]. Hollow silica nanoparticles (HSNs) refer to NPs with a solid shell, even though a recent trend has been to synthesize hollow nanospheres with a mesoporous silica shell. An advantage of the hollow form of MSNs is that their inner cavities can be used as containers for other cargo, such as magnetic, gold, or even smaller silica nanoparticles [83]. Hollow MSNs (HMSNs), a sub-class of MSNs with interstitial hollow space with extraordinarily high loading capacity, low density and high specific area, have been advocated as the new-generation of DDSs [63]. They are capable of holding a large amount of drugs in their hollow cavities, compared to their non-hollow counterparts. This unique property of HMSNs makes them widely useful in cancer therapy and imaging [1].

The development of novel fabrication methods for hollow MSNs has been one of the most active areas of research in nanotechnology. Numerous fabrication techniques exist to obtain hollow spheres with a wide range of diameters and wall thicknesses [80], including microemulsions, soft templating and hard templating [18]. However, the most frequently used approach for the synthesis of HMSNs is the core-templating method, where different soft and hard templates are used to form the nanostructure core that is then coated with the desired substance at different concentrations to obtain a shell around the substrate. After that, the template forming the core is removed by calcination or chemical etching, obtaining a shell with a hollow core [1]. Choosing the appropriate core template is critical for producing efficient hollow capsules, as their properties, such as size, shape and ease of removal, determine the properties of the final hollow capsules. Hollow nanocapsules with various diameters can, thus, be prepared by changing the diameter of these templates [84].

Hollow-type mesopores are created in the core-templating technique by exploiting the chemical differences between the core and shell of a silica core/mesoporous silica structure, in such a way that the core can be removed by heating (calcination) or by dissolution [80]. When an appropriate etching agent is used, a selective etching takes place at the interior, while the outer shell remains mostly intact and the hollow structure is formed [20]. The porous structure on the surface of the silica NPs provides a pathway for the etchant to penetrate the core of the silica material, thus facilitating further etching. Furthermore, when the silicate species reach the supersaturation level at the interface, condensation is expected to occur, leading to the expansion of the inner shell, while maintaining the overall shape [85]. There are some soft templates based on aggregates of organic molecules, including large micelles, vesicles and emulsions. Their structure and morphology can be easily controlled with the change of the preparative conditions, which enables various hollow-structured MSNs to be obtained. In the final step, they can be easily removed through calcination. Therefore, many hollow-structured MSNs have been prepared by using soft templates [83]. Tanev and Pinnavaia [86] condensed silica in the interlayer regions of multilamellar vesicles to form roughly spherical particles with stable lamellar mesostructures [87]. Using another approach, Lin et al. [88] prepared thermally stable NPs in a water-in-oil (*w/o*) emulsion, consisting of water, hydrocarbons and cationic surfactants [63].

The hard template method hollow-structured MSNs can be obtained with the selective elimination of the core particles [30]. Polymeric nanoparticles and carbon nanoparticles are some of the most frequently used hard templates, but metal oxide, metal nanoparticles and other inorganic nanoparticles have also been employed. Le et al. [45] described a synthesis pathway to obtain hollow silica spheres (40 nm average diameter) with mesoporous walls. The process was based on a double-template route using calcium carbonate nanoparticles as sphere templates and hexadecyltrimethylammonium bromide (C_16_TMABr) as a mesostructure-directing agent in alkaline conditions, obtaining silica hollow spheres at room temperature. The use of silica nanoparticles as templates (core particles) and coated with mesoporous silica shells has been widely reported. These silica cores present highly tunable characteristics, such as diameter, morphology and size distribution. The major problem of silica core nanoparticles is that separating the core from the silica shell requires a sophisticated procedure for selective core etching. For example, hollow organosiloxane NPs can be obtained by selectively etching the silica cores, thus exploiting the tolerance of organosiloxane toward base etching [30].

In the biomedical field, both discrete and monodispersed HMSNs play key roles in providing enough stability to the cargo in physiological environments and their nano-sizes enable effective distribution of the drugs in the body. Moreover, due to their hollow cavities, HMSNs have a large capacity to load biomedicines, enzymes, or small nanoparticles [63].

### 2.4. Other Silica Nanoparticles

While the development of silica NPs has historically been focused on MSNs, two additional types, mesoporous organosilica nanoparticles (MONs) and periodic mesoporous organosilica (PMO) NPs, are budding sectors in current silica NPs’ development, being one of the most representative inorganic–organic hybrid mesoporous nanoparticles. While PMO NPs and MONs are quite new, they already account for at least 10% of the papers currently being published on silica NPs [23].

As mentioned, when a single silica precursor, such as TEOS, is used, MSNs are formed. MONs will form by inserting organic groups into the framework of MSNs at the molecular level. The use of 100% of bridget-organosilane precursors, when no additional silane source is present, leads to the formation of PMO NPs [89,90]. Therefore, MONs are partially hybridized silica NPs, while PMO are exclusively formed by organically modified silicon atoms. This allows for the formation of a wide variety of additional silica nanoparticles with new chemistries, due to the presence of the organic (R) group.

As for MSNs, the most employed method is the sol-gel synthesis under basic or acid conditions, using surfactants to obtain the porous structure. When silanes and organosilanes ([(XO)_3_Si]_n_–R, where R is an organic group, n ≥ 1) are hydrolyzed in basic media, reactive silanolate species are produced. These species then condense with other (organo)silanes to create covalent siloxane linkages and progressively bigger oligomers. The sol-gel process eventually results in silica (SiO_2_) or silsesquioxane frameworks (e.g., O_1.5_Si-R-SiO_1.5_). Although some organically bridged structures are stable up to 580 °C, calcination is not likely to be a good alternative for MONs and PMO NPs, which is the method most commonly used to remove the surfactants from MSNs. Therefore, less aggressive extraction procedures are required [89].

MONs and PMO structures provide exponentially more design possibilities, as compared with MSNs, with the high potential of making uniquely functional silica nanoparticles, due to the flexibility of the organic R group, as previously mentioned [18]. Homogeneously distributed organic moieties throughout the mesoporous framework have a crucial impact on the size, the porosity and the morphology of the hybrid nanomaterials [91,92]. The R group can produce both physical changes to the pore size and shape and chemical changes affecting the hydrophobicity/hydrophilicity and charge of the particle have an impact on their final applications, such as the type of cargo that can be loaded on them or their loading capacities [23,93]. To add additional complexity, MONs have regions of MSNs and PMO that create hybrids, which can have different proportions of the organic and non-organic components, leading to the formation of unique porous structures [23]. Importantly, modifications of the R group allow for control over the biodegradability of these NPs, as will be described below in Section 5.2, being a key point for their biomedical application [90,94].

MONs and PMO NPs have been advocated as some of the most prosperous nanomaterials over the next decade for their application in multiple fields, such as catalysis, gas and molecule adsorption, electronics, drug and gene delivery, biosensing, photodynamic therapy, or molecular imaging, among others [23,93].

Despite the benefits that MON and PMO NPs offer, their use in drug delivery has not received as much research attention as that of MSNs of pure silica. This could account for the difficulty in synthesizing uniform and discrete NPs with adjustable properties and the lack of knowledge regarding their biosafety [17,95]. In addition, despite the potential of MONs and PMO NPs for medical applications, they will encounter a larger barrier than MSNs to reach the clinic. This is because toxicological studies on MSNs do not translate over to MONs or PMOs, and each R group arrangement would require further toxicological testing. Thus, although FDA considers silica as safe, MONs and PMO NPs are not benefited by this because they are regarded as brand new compounds [23,89].

## 3. Applications of MSNs

Numerous research groups have employed monodispersed silica colloids in a diversified range of applications. It is always preferable to use silica particles with specific particle size and narrow size distribution [14], hence the interest of the previous section. MSNs have several potential applications, depending on their size, shape, connectivity and the nature of their pores. Sacks and Tseng [96] used silica colloids to pack ordered structure membranes and investigated their sintering behavior. Unger et al. [97] applied the submicron silica colloids as packing material for capillary chromatography. Míguez et al. conducted numerous studies on the use of monodispersed silica colloids to fabricate photonic crystals of 3D periodic structures [98]. Due to its regular pore structure, pore shape and large internal surface area, MCM-41 has attracted considerable interest [38]. MCM-41 may find significant use as an adsorbent, due to its hydrophobic and hydrophilic characteristics, which vary depending on the exact composition and/or post-synthesis modifications. Consequently, the removal of hydrocarbons from water, storage of gases (H_2_, O_2_, CH_4_, etc.) and separation of biological and pharmaceutical compounds have been deemed as potential areas for the application of MCM-41. MCM-41 may also be useful in the development of environmentally safe processes, including the replacement of environmentally hazardous catalysts. The catalytic properties of MSNs can be adjusted by changing the surface morphology to incorporate different metal oxides and metal complexes into the MCM-41 framework [63]. In addition, metal and metal complexes encapsulated within the internal surfaces of MCM-41 and offer significant promise as potential alternatives to conventional heterogeneous catalysts, due to their ready regenerability, shape selectivity, easy separation and recovery [36]. An emerging area of research that is obtaining increased attention focuses on the use of silica scaffolds, including MSNs, for the creation of nanorobots [99,100]. From the wide variety of applications of MSNs, we will focus on their use in the biomedical field and, in particular, as drug delivery systems and for biomedical imaging. This is because their combination allows for the use of these versatile nanosystems in the fast-evolving field of theranostics. The presence of an imaging and therapeutic agent in the same nanoparticle allows not only for ensuring the delivery of the therapeutic agent at the targeted site, but also for monitoring the therapeutic effect.

### 3.1. MSNs in Biomedical Applications

MSNs contain small channels that improve the transport of biomolecules. As a result, these materials are considered excellent carriers for drug delivery, due to their specificity, dispersibility and capacity to load and deliver a high concentration of different molecules. Their textural properties favor their use for the treatment of various diseases, including diabetes, inflammation and cancer, as well as in bone/tendon tissue engineering [15]. The biological applications of MSNs also include their use as imaging and diagnostic agents. MCM-41 was the first mesoporous silica material to be reported as a DDS. Since then, MSNs have been identified as promising nanocarriers for the transport of highly toxic drugs, such as chemotherapeutic agents with site-specific characteristics, for the purpose of selectively killing tumor cells [63]. Additionally, the drug diffusion kinetics can be controlled via the functionalization of the silanol groups on the external surface and the interior pores of MSNs. The functionalization might also facilitate the loading of organic molecules, including not only therapeutic, but also imaging agents [63].

The rational delivery and targeting of therapeutic and diagnostic agents is at the forefront of cancer research [101]. Cancer is one of the leading causes of death globally, especially in western countries, posing an enormous challenge, in terms of prevention and treatment. According to the International Cancer Observatory, roughly 19 million people have developed cancer and 9.9 million have died, in 2020 [102]. Cancer is commonly defined as an uncontrolled proliferation and development of cells in tissues, forming tumors that may potentially create metastasis by their expansion to a whole organ or systemically to other tissues [103]. Recent advances in nanotechnology have offered new opportunities for cancer prevention, diagnosis and treatment [104,105]. Thus, novel nano-scale materials are being proposed for use as potential drug delivery systems, diagnostic markers and contrast agents in medical imaging [106,107]. One of the most significant challenges in nanotechnology-based cancer targeted therapy is achieving sufficiently high drug concentrations in tumor locations and minimizing accumulation in the healthy tissue, thus improving the therapeutic efficiency and reducing side effects. Thus, the biodistribution, metabolism and clearance of these nanoparticulate DDSs determine the final therapeutic index. Additionally, it is defined by their capacity to cross biological barriers and penetrate and accumulate in tumor tissues [18]. Chemotherapy currently requires high doses of cytotoxic drugs, due to their lack of specificity, which results in severe cytotoxic effects on healthy tissues and organs and, consequently, results in severe adverse effects in the patient. Conventional antitumor drugs are small and can pass through blood vessels in tumors and healthy tissues. As a result, they are rapidly eliminated from the bloodstream, necessitating high doses to achieve therapeutic levels [108]. While some nanoparticles exhibit acute toxicity, the therapeutic dose for MSNs delivery is well below the toxic dose, making them an excellent choice for medical applications [23,109].

Even though, to the best of our knowledge, there are no ongoing clinical trials with MSNs to date, a pilot study was performed in humans with ordered mesoporous silica [110]. An enhancement in the bioavailability of a drug with poor solubility (fenofibrate) was observed after oral administration in the absence of serious adverse events. On the other hand, other silica formulations reached clinical trials; some of them are currently ongoing and others have been already completed (NCT02106598, NCT04167969, NCT01436123). Silica nanoparticles (core) with a gold shell, Auroshells, have already shown successful results in head, neck and prostate cancer patients in clinical trials [111] (NCT00848042, NCT04240639, NCT02680535 and NCT04656678). Ultrasmall silica nanoparticles (Cornell dots), also tested in clinical trials (NCT03465618, NCT01266096 and NCT02106598), have shown feasibility and safety in humans [112]. At the micro-scale, radioactive silica microspheres (TheraSpheres^TM^) have been approved for their use in humans for the treatment of liver cancer, showing the potential of the silica-based materials to be translated to the clinic [113]. One of the current scientific goals is to develop targeted smart drug delivery nanosystems capable of effectively delivering a drug dose to target cells [43].

#### 3.1.1. MSNs as Drug Delivery Systems

Among all available nanomaterials and inorganic structures, porous silica materials, with their tailored mesoporous structure and high surface area, comprise one of the most promising nanocarriers in drug delivery, since they exhibit significant advantages over traditional delivery systems [2]. The promising properties of MSNs for drug delivery applications can be clearly appreciated by the large amount of research articles that have been recently published in this area. Some selected examples are included in Table 1, reflecting on the versatility of MSNs to transport diverse drugs and treat multiple diseases. Using conventional and new DDS, they are expected to overcome the main problems in drug delivery, including unfavorable pharmacokinetics and biodistribution, which lead to undesired side effects, premature drug degradation in the blood and inefficient uptake at targeted sites, which lead to low drug efficacy. Currently, only a small number of nanocarriers are capable of achieving this feat [70]. The aim of such nanocarriers is to encapsulate, chemically attach, or adsorb therapeutic agents to the nanoparticles to overcome drug solubility problems. Functionalization of their surface with antibodies or appropriate ligands allows for the use of nanoparticles for targeting specific cells and tissues within the body [101]. Since the initial studies in the early 2000s by Vallet-Regí et al. using MCM-41 and ibuprofen as the model drug [59], an exponential increase in research on MSNs as DDS has been witnessed [17]. Although most nanocarriers have been primarily used for single drug delivery, mesoporous silica materials are considered excellent carriers for drug delivery, due to their morphology and unique properties, which facilitate the introduction of a number of therapeutic agents into the pores [114]. The large amount of pores and the high surface area define MSNs as a potential multifunctional theranostic agent. They are especially appropriate for incorporating the essential capabilities of a theranostic platform in a single particle, with separate domains for the contrast agent (that enables traceable imaging), the drug payload (for therapeutic intervention) and the biomolecular ligand (for highly targeted delivery). Moreover, the adjustable pore size allows us to improve the control over the drug loading and release kinetics. The therapeutic efficacy can be also improved, thanks to modifiable surface for controlled and targeted drug delivery, at the same time decreasing their toxicity. In addition, the combination with magnetic and/or luminescent compounds enables simultaneous drug delivery and bioimaging [106]. MSNs presents notable biocompatibility and delivery abilities, but their distinctive point with other carriers is their flexible platform that endows the system with a range of potential functionalities and modifications. Lastly, they are easy to produce, being interesting in terms of practicality, as with the new synthetic processes and the abundance of silica precursors MSNs can be produced at low costs and in large quantities [70,115].

All these advantages have led functionalized MSNs to represent a feasible alternative therapy for multiple and diverse diseases that lack effective treatment nowadays. Thus, drug delivery by MSNs has shown promising preclinical results in vivo not only for cancer (including melanoma [116], pancreas [117], bladder [118], breast [119] and osteosarcoma [120], among others), but also myocardium infarction [121], cartilage [122] and bone regeneration [123,124], neurodegenerative diseases, such as ALS [125] or Alzheimer [126] and infection prevention with vaccines [127], to name some.

While MSNs inherently have a greater loading capacity than other carriers, due to their large pore volume, there is a way to improve MSNs loading. As already mentioned, hollow MSNs (HMSNs), thanks to their hollow cavities, have been demonstrated to be a better carrier, in terms of loading capacity, as 3–15 times higher loading of drugs was observed when compared to MSNs [1].

#### 3.1.2. MSNs in Biomedical Imaging and as Theranostic Agents

In the field of nanomaterials and nanobiomedicine, numerous multifunctional MSNs have been extensively studied, due to the advantageous chemistry of silane groups. The interior pores of MSNs facilitate the loading of MRI contrast agents, fluorescent molecules and positron emission tomography (PET). These can be combined with drugs, which, as previously discussed, hold great interest for theranostic applications, as has been mentioned before [128,129]. Table 2 summarizes some of the most recent studies that develop MSN platforms for bioimaging or for theranostic applications.

**Table 1:** Selected recent examples of MSNs developed as drug delivery systems and evaluated in biological models. Sample names used by the authors are included, since, in some cases, they provide information about the composition of the nanocarrier. EDTA: EthyleneDiamineTetraacetic Acid. MSN: Mesoporous Silica Nanoparticle. NA: Not Available. PEG: PolyEthylene Glycol.

Aimed Disease or Condition	Sample Name	Size	Targeting and/or Triggered Release	Surface Modification (s)	Therapeutic Agent (s)	Biological Model (s)	In Vivo Administration Route	Reference
Gastrointestinal oral absorption efficiency	FFB-MSNs	Diameter: 130 nm	Passive targeting	-	Fenofibrate (FFB)	In vitro drug release In vivo rats (Sprague Dawley strain): pharmacokinetics and intestine uptake and retention	Oral (water)	[130]
		W: 65 nm; L: 185 nm						
		W: 50 nm; L: 23 nm						
Gastrointestinal oral absorption efficiency	IMC–MSNs@HPMC	~60 nm	Passive targeting	-	Indomethacin	In vivo rats (Sprague-Dawley): pharmacokinetics and biodistribution	Oral	[131]
	IMC–MSNs@Kollicoat IR							
Amyotrophic Lateral Sclerosis (ALS)	MSN-LEP-PIO	~94 ± 15 nm.	Passive targeting	(3-Aminopropyl)triethoxysilane (AP), leptin and pioglitazone	Drug cocktail: Leptin (LEP) and Pioglitazone (PIO)	In vitro drug release	Intraperitoneal	[125]
						In vivo mice (transgenic, TDP-43 proteinopathy (TDP-43^A315T^ mice): functional evaluation biodistribution.		
Alzheimer	MSN-CCM	60 nm	Thermo-responsiveness	-	Curcumin (CCM)	In vitro ex vivo mucoadhesion and permeation studies, cytotoxicity	Hydrogel: oral; intranasal	[126]
						In vivo mice (Swiss albino): behavioral assessment		
Blood–brain-barrier crossing	MSN-TQ	90 nm	Passive targeting	-	Thymoquinone (TQ)	In vitro drug release	Intraperitoneal	[132]
						In vivo rats (SD): biodistribution, oxidative and non-oxidative stress parameters (GST, GSH, NO)		
Miocardium infarction	MSN-NGR1-CD11b	83 nm	Active targeting (CD11b)	-	Notoginsenoside R1 (NGR1)	In vitro cytotoxicity assay, ROS generation, oxidative DNA damage assessment, apoptosis assessment	Intragastric, intravenous.	[121]
						In vivo mice (BALB/c nude, C57BL/6J; Zsgreen transgenic), myocardium infarction model: biodistribution, toxicity		
Liver fibrosis	IMB16-4-MSNs	~60 nm	Passive targeting	-	IMB16-4 (N-(3,4,5-trichlorophenyl)-2(3-nitrobenzenesulfonamide) benzamide)	In vitro drug release, cytotoxicity, antifibrotic effect evaluation	Oral	[133]
						In vivo rats (Sprague-Dawley); pharmacokinetics		
Bone regeneration in osteoporosis	NaLuF_4_:Yb,Tm@NaLuF4@mSiO2-EDTA-E_2_	20 nm	Active targeting (EDTA)	EDTA	17β-estradiol (E_2_)	In vitro cytotoxicity, cellular uptake, alkaline phosphatase and alizarin red S staining	Intravenous	[133]
	(E_2_-csUCNP@MSN-EDTA, UCHRT)					In vivo mice (Kunming mice): biodistribution, mechanical assessment, bone turnover assessment		
Periodontal bone regeneration in diabetes mellitus	PPP-MM-S	~ 50 nm	Thermo-responsiveness (PDLLA-PEG-PDLLA)	PDLLA-PEG-PDLLA	SDF-1	In vitro osteogenesis assessment, ROS generation, alkaline phosphatase and alizarin red S staining, migration assay, cytotoxicity, protein profile assessment	Hydrogel	[123]
	(PDLLA-PEG-PDLLA-Met@MSN-SDF-1 )					In vivo rats (Sprague-Dawley): degradation assessment, toxicity, bone regeneration assessment		
Vascularization for bone repair	S1P@MSNs/ALG/NOOC	~150 nm	Passive targeting	Alginate (ALG) and chitosan (CHI)	aldehyde hyaluronic acid (AHA) and N,O-carboxymethyl chitosan (NOCC)	In vitro degradation, drug release, cytotoxicity, migration assay, chorioallantoic membrane assay	Topic	[134]
						In vivo mice (ICR): vascularization assessment		
Antiangiogenic therapy	Bevacizumab-MSN	140 ± 18 nm	Active targeting (vascular endothelial growth factor receptor, VEGFR)	PEG	Bevacizumab	In vitro drug release, cytotoxicity, tube formation assay	Intravitreal	[135]
	MSN-encapsulated bevacizumab					In vivo mice (C57BL/6J): pharmacokinetic evaluation, neovascularization assay		
Hepatitis C infection	VLP-MSNs	186 nm	Passive targeting	-	Velpatasvir	In vitro drug release, cytotoxicity	Oral (food)	[136]
						In vivo rats (Sprague-Dawley): pharmacokinetics, toxicity		
Hemorrhage	MSN–GACS	~60 nm	-	-	glycerol-modified N-alkylated chitosan sponge (GACS)	In vitro hemostatic efficiency assays, hemolytic test, cytotoxicity	Topic (gauze)	[137]
						In vivo rats (Sprague-Dawley), liver injury model and prognosis; rabbits (New Zealand), femoral artery injury model		
Wound infection	AMPC@siTNF-α	~100 nm	-	PEG, CFL, siTNF-α	Silver (Ag^+^), Ciprofloxacin (CFL) and Tumor Necrosis Factor-α (TNF-α) small interfering RNA (siTNF-α)	In vitro drug release, cytotoxicity, hemolysis, anti-inflammatory assay, anti-bacterial activity assessment In vivo mice (BALB/c): wound healing assessment, safety	Topic	[138]
Infections and cancers	MSNs@Cy7-PA-C1b@FA-GO	100–120 nm	Active targeting (folic acid)	Graphene oxide (GO), folic acid (FA)	Antimicrobial peptide PA-C1b (chensinin-1b conjugated with palmitic acid)	In vitro drug release, intracellular localization assessment	Intratumor	[139]
			Light-mediated peptide release			In vivo mice (nude): biodistribution; anticancer activity		
Cancer (melanoma)	CP/CQ@MSN−HtB/Cu^2+^	160 nm	Active targeting (HtB)	Histidine-tagged targeting peptide (HtB) and Cu2 (pore sealing)	Cisplatin (CP) and chloroquine (CQ)	In vitro drug release assay, cytotoxicity, cellular uptake, intracellular ROS generation	Intravenous	[116]
			pH-responsiveness			In vivo mice (C57BL/6), subcutaneous model: biodistribution, antitumor efficacy and biosafety		
Cancer (pancreas)	CyP-MSNs	252 ± 40 nm	Passive targeting	PEG	Cyclopamine (CyP), gemcytabine (Gem), cisplatin (cisPt)	In vitro cytotoxicity, pathway inhibition assay	Intratumor, intravenous	[117]
	PEG-Gem-cisPt-MSNs					In vivo mice, subcutaneous model: antitumor effect		
Cancer (bladder)	c(RGDfK)-MSN NPs	100–200 nm	Active targeting (RGDfK)	PLGA-PEG decorated with c(RGDfK)	miR-34a and siPD-L1	In vitro siRNA/miRNA release, cellular uptake, cytotoxicity, migration and invasion assays, target inhibition, protein expression	Intravenous	[118]
						In vivo mice, intraperitoneal model: antitumor effect, toxicity assay		
Cancer (breast)	MSN-Res	~60 nm	Passive targeting	-	Resveratrol (Res)	In vitro cytotoxicity, cell migration and invasion assays, annexin-V assay	NA	[119]
						In vivo mice (BALB/c nude), subcutaneous model: antitumor effect, toxicity assay		
Cancer (osteosarcoma)	MPCT@Li-R NPs	95 nm	Active targeting (RGD)	Liposome shell; RGD	Photosensitizer chlorin e6 (Ce6) and MTH1 inhibitor TH588	In vitro drug release assay, cytotoxicity, hemolysis assay, cellular uptake, ROS generation assay	Intravenous	[120]
	MSN-Pt NPs					In vivo mice (BALB/c nude), subcutaneous model: biodistribution, antitumor therapy, toxicity		
Cancer (lung)	MSN@OHA-Ce6/BSO/Pt	∼211.4 nm	pH-responsiveness	Oxidized hyaluronic acid (OHA) as pore-blocking agent. + Cisplatin (Pt), Chlorin e6 (Ce6)	Buthionine-sulfoximine (BSO), chlorin e6 (Ce6) and Cisplatin (Pt)	In vitro drug release assay, cellular uptake, ROS generation, cytotoxicity	Intravenous	[140]
						In vivo mice (nude), subcutaneous model: biodistribution, biocompatibility, antitumor		
Cancer (gastric)	MSN/Res-PEI-FA	100 nm	Passive targeting	Polyethylenimine (PEI), folic acid (FA)	Resveratrol (Res)	In vitro cytototoxicity, apoptosis assay, migration and invasion assays	NA	[141]
						In vivo mice (BALB/c nude) SC: antitumor effect and toxicity		
Solid tumors	1j@-MSN-PBA-GN	~86 nm	Active targeting (phenyl boronic acid, PBA)	PBA	1j (synthetic compound)	In vitro intracellular drug release and uptake, cytotoxicity; ROS generation and mitochondrial membrane potential	Intravenous	[142]
			Redox-responsiveness			In vivo mice (Swiss albino mice): biodistribution, antitumor effect		
Cancer (breast)	DOX@MSN-CHI-RGD-PEG	~155 nm	Active targeting: adamantane-glycine-arginine-glycine-aspartic acid-serine (Ad-GRGDS)	mPEG	Doxorubicin hydrochloride (DOX)	In vitro stability, drug release, cytotoxicity, antitumor assays, cellular uptake	Intravenous	[143]
			pH-responsiveness			In vivo mice (BALB/c), orthotopic model: antitumor, toxicity		
Cancer	DOX-loaded MSN@M	~ 90 nm	Active targeting (mesenchymal stem cells membrane)	Mesenchymal stem cells membrane (M)	Doxorrubicine (DOX)	In vitro drug release, cellular uptake, cytotoxicity, immune escape capacity	Intravenous	[144]
						In vivo mice (BALB/c nude; ICR), subcutaneous model: parmacokinetics, biodistribution, antitumor effect, biocompatibility		
Cancer (melanoma)	MSN(Mn)-ICG/DTIC	125.57 ± 5.96 nm	Photothermal activation	-	Dacarbazine (DTIC) and indocyanine green (ICG)	In vitro cytotoxicity, chemo-phototherapy, apoptosis assay	Intratumor	[145]
						In vivo mice (BALB/c nude), subcutaneous model: antitumor effect		
Cancer (liver)	TLS11a-LB@TATp-MSN/DOX	100 nm	Active targeting: Liver cancer-specific aptamer (TLS11a-LB) and nuclear targeting (TATp)	Lipid bilayer, PEG.	Doxorrubicine (DOX)	In vitro drug release, intracellular localization, cytotoxicity	Intravenous	[146]
						In vivo mice (BALB/c), subcutaneous model: biodistribution, antitumor effect		
Cancer (colon)	PEG@MSNR-CPT PEG@MSNR-CPT/Sur Apt-PEG@MSNR-CPT/Sur	~250 nm ~150 nm ~150 nm	Active targeting (nucleolin)	APTES, PEG	Camptothecin and survivin shRNA	In vitro drug release, cytotoxicity, apoptosis assay	Intravenous	[147]
						In vivo: C26 tumor bearing mice: biodistribution, antitumor effect		
Cancer (breast)	Umbe@MSN-PAA-FA	40–50 nm	Active targeting (folic acid)	Polyacrylic acid (PAA), folic acid (FA)	Umbelliferone (Umbe)	In vitro drug release and intracellular uptake; cytotoxicity, ROS generation, MMP determination	Intravenous	[148]
			pH responsiveness (PAA)			In vivo mice (Swiss albino): biodistribution, antitumor effect, toxicity systemic evaluation		
Solid tumors	CMSN-PEG	150 nm	Passive targeting	PEG	Celastrol (mitochondrial targeting)	In vitro drug release, cytotoxicity, cellular uptake, apoptosis assessment	Intravenous	[149]
						In vivo mice (BALB/s nude), subcutaneous model: antitumor effect and toxicity		

**Table 2:** Selected recent examples of MSNs developed as biomedical imaging or theranostic agents and evaluated in biological models. Sample names used by the authors are included, since, in some cases, they provided information about the composition of the nanocarrier. MSN: Mesoporous Silica Nanoparticle. MRI: Magnetic Resonance Imaging. NA: Not Available PEG: PolyEthylene Glycol. PET: Positron Emission Tomography. SPECT: Single-Photon Emission Computed Tomography. CT: Computerized Tomography.

Imaging Modality	Aimed Disease or Condition	Sample Name	Size	Targeting and/or Triggered Release	Surface Modification (s)	Imaging (and Therapeutic) Agent	Biological Model (s)	In Vivo Administration Route	Reference
MRI	Cancer	SA-Gd_2_O_3_@MSN	83.2 ± 8.7 nm	pH-responsiveness	Sodium alginate	Gadolidium (Gd) for MRI	In vitro hemolysis assays, cellular uptake	Intravenous	[150]
		Rhodamine B					In vivo mice (BALB/c): biodistribution		
MRI	Cancer	HA-MnO@MSN	50 nm	Active targeting (CD44)	Hyaluronic acid (HA)	Mn^2+^ for MRI	In vitro MRI imaging	Intratumor	[151]
							In vivo mice, subcutaneous model: MRI imaging, tumor uptake		
MRI	Atherosclerosis	cRGD-platelet@MnO/MSN@PPARα/LXRα	~150 nm (DLS)	Active targeting (cRGD to integrin αvβ3)	cRGD-platelets	MnO for MRI PPAR and LXRα as therapeutic agent	In vitro cytotoxicity, ROS generation,	Intravenous	[152]
							In vivo rats (Sprague Dawley): MRI imaging, apoptosis assessment, ROS generation, biodistribution, toxicity		
MRI	Inflamation	Fe/Ce-MSN-PEG	190 ± 1.2 nm (DLS)	Passive targeting	PEG	Mn, Fe for MRI	In vitro MRI assay, cytotoxicity, cellular uptake, anti-apoptotic activity assay, ROS scavenging assay, anti-inflammation assessment	-	[153]
				pH responsiveness					
MRI	Cancer	MN@MS@CS@ABE	131 ± 18 nm	Passive targeting	Chitosan (CS)	Abemaciclib (ABE) as therapeutic agent Magnetite Nanoparticles (MN) for MRI	In vitro MRI assay, drug release, cytotoxicity, cell cycle and apoptosis assessment	-	[154]
MRI	Cancer (breast)	MSN-Ce6@PDA (Mn)	139 ± 1.70 nm	PDA as photothermal agent	Polydopamine (PDA)	Mn^2+^ for MRI	In vitro biocompatibility, cytotoxicity, cellular uptake, in vitro MRI, ROS generation	Intravenous	[155]
							In vivo mice (BALB/c nude), subcutaneous model: biodistribution, MRI imaging, antitumor effect, toxicity		
MRI	Cancer	GdBO_3_ @mSiO_2_-PG	~100 nm	Passive targeting	Hydrophilic polyglycerol (PG)	GdBO_3_ for neutron capture therapy and MRI	In vitro cytotoxicity, cellular uptake	Intravenous	[156]
							In vivo mice (BALB/c): blood circulation assessment, biochemistry examinations, toxicity		
MRI	_	Gd_2_O_3_@MSN	86.85 ± 10.44 nm	Passive targeting	-	Gd_2_O_3_ for MRI	In vitro cytotoxicity, MRI	Intravenous	[157]
							In vivo rats (Spraque-Dawley): toxicity, MRI imaging		
MRI	Kidney disfunction	Gd@PEG NPs	~5 nm	Passive targeting	PEG	Gadolinium (Gd) for MRI	In vitro cytotoxicity	Intravenous	[158]
							In vivo mice: imaging, biodistribution, toxicity		
MRI and phototermal imaging	Cancer (melanoma)	MSN(Mn)-ICG/DTIC	125.57 ± 5.96 nm	Photothermal activation	-	Mn^2+^ ions for MRI Indocyanine green (ICG) for photothermal imaging	In vitro cytotoxicity, chemo-phototherapy, apoptosis assay	Intratumor	[145]
							In vivo mice (BALB/c nude), subcutaneous model: antitumor effect.		
MRI Optical imaging	Atherosclerosis	PP1-IO@MS-IR820	90 nm	Active targeting (PP1, towards macrophages)	PEG, PP1	Iron oxide (IO) for MRI IR820 for NIR optical imaging	In vitro MRI and fluorescence imaging, cytotoxicity, target assessment	Intravenous	[159]
							In vivo mice (ApoE^−/−^); in vivo MRI imaging, ex vivo fluorescence imaging, toxicity		
MRI Optical imaging	Cancer (prostate)	PSA-Mn-Msn-Cy7	50 nm	Active targeting (PSA)	DSPE-PEG2000-COOH	Cy7 for optical imaging Mn^2+^ for MRI	In vitro cytotoxicity, in vitro MRI and TEM	Intravenous	[160]
							In vivo mice (nude), subcutaneous model: toxicity, pharmacokinetics, imaging, Mn determination		
Optical imaging	Cancer	DCNPs@Si-omSi	∼255 nm (DLS)	Active targeting (RGD to integrin αvβ3)	DSPE-PEG_2000_-NH_2_	Indocyanine green (ICG) for imaging	In vitro fluorescent dye stability, cellular uptake by flow cytometry and microscopy	Intravenous	[161]
							In vivo: mice (BALB/c) subcutaneous model for imaging guided tumor surgery		
Optical imaging	Cancer	PPV@MSN-CP1@FA	~100 nm	Active targeting (folate acid, FA)	DSPE-PEG2000, folic acid (FA)	PFV-co-MEHPV (CP1) for fluorescent imaging of ROS	In vitro confocal laser scanning microscopy imaging, cellular uptake	Intratumor	[162]
							In vivo mice (BALB/c), subcutaneous model: imaging		
Optical imaging PET	Cancer (breast)	NOTA-QD@HMSN(DOX)-PEG-TRC105	~ 72 nm	Active targeting (CD105)	SCM-PEG5k-Mal, NOTA, TRC105	QDs for optical imaging	In vitro targeting assay	Intravenous	[163]
		^64^Cu-NOTA-QD@HMSN-PEG-TRC105				^64^Cu for PET imaging	In vivo mice (BALB/c), subcutaneous model: biodistribution, toxicity		
PET	Atherosclerosis	^18^F-DBCOT-MSNs	≈ 60–80 nm	Passive targeting	PEG, azadibenocyclooctyne (DBCO)	^18^F for PET imaging	In vitro proliferation, cytokine assay, cell uptake, phagocytic activity assay, macrophage cell labeling	Retro-orbital	[164]
							In vivo mice (nude; ApoE^−/−^); MSNs-labeled macrophage cell tracking, imaging, toxicity		
PET	Cancer	*As-MSN	65 ± 5 nm	Passive targeting	Thiol functional groups, radioarsenic	Radioarsenic for PET imaging	In vitro nanoparticle stability	Intravenous	[165]
		[*As]ATO-MSN	150 ± 5 nm			Arsenic trioxide (ATO) as therapeutic agent	In vivo mice (BALB/c): nanoparticle stability, biodistribution		
Multimodal NIR-PL/MR/PET	Cancer	^68^Ga /DOX/Si-Pc-Loaded HMNPs	∼93–98 nm	Passive targeting NIR-PL-sensitized photodynamic therapy	-	^68^Ga for PET imaging Ga_2_O_3_:Cr^3+^, Nd^3+^ for NIR-PL imaging Gd_2_O_3_ for MRI Si-Pc as photosensitizer Doxorubicin hydrochloride (DOX) as therapeutic agent	In vitro nanoparticle stability, imaging drug release, cellular uptake, cytotoxicity, hemolysis	Intravenous	[166]
							In vivo mice (BALB/c nude), subcutaneous model: MRI imaging, PET imaging, NIR-PL im aging, chemotherapeutic effect, photodynamic therapy, toxicity		
Multimodal: PET and photoacoustic imaging	Cancer	^89^Zr-labeled bGNR@MSN(DOX)-PEG	L: 104.6 ± 5.6 nm	Passive targeting	PEG	Doxorubicine (DOX) as therapeutic agent	In vitro drug release, photothermal and chemo-photothermal effect assessments	Intravenous	[167]
			W: 68.6 ± 5.2 nm			^89^Zr for PET imaging	In vivo mice: PET and PA imaging; antitumor effect		
SPECT/CT		PEG-PEI-*In- MSNs PEG-QA-*In- MSNs PEG-TMS-*In- MSNs	32 ± 1 nm 55 ± 1 nm 93 ± 1 nm 142 ± 1 nm 52 ± 2 nm 56 ± 2 nm	Passive targeting	PEG-polyethylenimine (PEG-PEI) PEG-quaternary amine (PEG-QA) PEG-trimethylsilane (PEG-TMS)	^111^Indium for SPECT imaging	In vitro nanoparticle stability	Intravenous Intraperitoneal	[168]
							In vivo rats (Fischer 344): SPECT/CT imaging, biodistribution, pharmacokinetics		

##### Optical Imaging

Optical imaging (OI) is a technique wherein specific probes are excited by incident light, usually in the visible or near-infrared regions, resulting in the emission of light at a lower energy level. Some of the major drawbacks of many fluorophores, especially the near-infrared (NIR) ones, is their poor solubility and stability. To overcome these problems, they can be loaded into MSNs, improving their photophysical and photochemical properties [1]. MSNs are optically transparent, since their nano-scale particle sizes do not absorb light in the near-infrared (NIR), visible and ultraviolet regions. Consequently, MSNs would not disturb the emission of fluorescent agents. Their hydrophilic surface also allows for the proper dispersion of the fluorescent agent, upon loading. Thus, MSNs have been modified with various fluorescent dyes for bioimaging applications in numerous studies [106]. There are different methods for fluorescently labeling MSNs. Fluorescent dye molecules can be attached to the particles, in both the inner and outer surfaces, by different strategies, depending on if the labeling has to be removable or not, including covalent linkage via post-synthetic grafting, incorporation into the silica framework, or pH-/redox-sensitive linkage. Optical imaging is highly used to study the feasibility of custom-made drug nanocarriers, based on MSNs. Fluorescent microscopy can track the direct release and distribution of cargo inside cells. Due to the non-invasive nature of optical microscopy, their use in combination with NIR fluorescent dyes, which can be detected after intravenous injection, enables the investigation of the long-term biodistribution of nanocarrier systems [169], as has been proven in in vivo models of breast and prostate cancer [160]. Furthermore, modified MSNs can also serve as nanocarriers of activatable fluorescent particles to optically detect physiological environment conditions, such as hypoxia, as studied in an in vivo model of inflammatory bowel disease, representing an interesting tool for the precise diagnosis and treatment of this disease [170].

##### Magnetic Resonance Imaging

Magnetic resonance imaging (MRI) is a powerful in vivo imaging technique that provides a three-dimensional anatomical picture of the region of interest with a high resolution [1]. A complementary technique, contrast-enhanced MRI, enables the quantitative assessment of disease pathogenesis through the measurement of up-regulated biomarkers. A variety of compounds, mainly those based on gadolinium chelates, are currently used as MRI contrast agents. However, these molecules lack sensitivity and often do not provide satisfactory image contrast enhancement in early disease stages. Therefore, alternative contrast agents are being explored. Due to their high surface areas and adaptable pore structures, mesoporous materials are an ideal platform for the development of hybrid materials for magnetic resonance imaging. There is a vast amount of research in this area, as can be appreciated by the recent studies that focus on the synthesis of MSNs for MRI (Table 2) [171]. Consequently, MSNs containing gadolinium (Gd) chelates, iron oxide, or manganese oxide NPs have been tested as potential MRI nanostructures [106]. MSNs with magnetic NPs have been employed to simultaneously induce the controlled release of payloads and serve as MRI agents. The incorporation of magnetic NPs allows for the use of the functionalized MSNs-based nanocarriers as non-invasive MRI agents for the real-time monitoring of the tumor treatment process in living animals [40]. MSNs have also been used in conjunction with both the OI and MRI imaging modalities [172]. OI is a relatively low-cost technology that allows for rapid screening and MRI can offer high resolution, while simultaneously obtaining physiological and anatomical information [43]. In vivo, MSNs loaded with multimodal imaging contrast agents for cancer have demonstrated a highly translational potential, not only regarding the enhancement of tumor optical or MRI imaging, especially when targeted [160], but also as therapeutic agents by photothermic and photodynamic combination [155] or as oxygen modulators [151].

##### Positron Emission Tomography

Positron emission tomography (PET) takes advantage of radionuclides that emit positrons during their decay. The positrons then react with the electrons nearby, annihilating into two distinct gamma rays in opposite directions, which can then be detected by a scanning device [23]. PET is a non-invasive technique that allows for a quantitative evaluation of the biodistribution of the particles in vivo, as it has a superior signal tissue penetration and sensitivity. The major problem is that the spatial resolution is lower, compared with MRI, usually being employed along with other imaging techniques, such as computed tomography (CT) and MRI. The functionalization of mesoporous nanomaterials with radionuclides leads to the higher selectivity and intensity of the PET signal in vivo, compared to free radioisotopes [89]. It is possible to improve the tissue selectivity and the PET resolution by loading MSNs with radionuclides, such as zirconium-89, arsenic-72, copper-64, technetium-99, titanium-45, fluorine-18, or carbon-11, due to the high overall signals. The loading of the radionuclides into the MSNs allows us to protect them from being lost during systemic circulation and increase their accumulation in the target tissues [173]. Thus, MSNs provide a novel way of improving the specificity and resolution of PET [24,91,109]. This has already been exploited in the preclinical phases in mice, in an attempt to elucidate their biodistribution, regarding their physicochemical properties and route of administration [165,168]. Furthermore, radiolabeled MSNs arise as potent theranostic alternatives or including multimodal imaging, as shown in mice cancer models [166].

#### 3.1.3. MSNs in Vaccines

For more than two centuries, vaccinations have been at the vanguard of advancing human health. The difficulties in creating effective vaccines originate from the complexity of the immune system and the need for a powerful adaptive immunological response, which calls for an effective and secure adjuvant [174]. Adjuvants are substances that are added to vaccination formulations to act as delivery mechanisms to help antigen uptake or to have an immunostimulatory effect on the antigen-presenting cells (APCs).

Many efforts have been made over the years to create vaccinations that are long-lasting, non-invasive and thermoresistant, and require few or no booster doses. Despite many years of development, only a few types of adjuvants are currently approved for human use, such as aluminium-based mineral salts, virus-like particles, oil emulsions and bacterial derivatives [175]. As a result, extensive research has concentrated on creating novel adjuvants, such as inorganic salts, proteins, oils, membrane complexes, or nanostructured materials [175].

The application of nanotechnology in vaccination has grown rapidly, giving rise to the field of “nanovaccinology” in both prophylactic and therapeutic approaches [176]. The use of nanoparticles in vaccine formulations allows not only improved antigen stability and immunogenicity, but also targeted delivery and slow release [175,176]. As delivery systems, nanoparticles can deliver antigens to the cells of the immune system or act as transient delivery systems, protecting the antigens until their release at the target location. In immune potentiation strategies, nanoparticles activate certain immune pathways, which might then enhance antigen processing and improve immunogenicity. Several nanocarriers have been evaluated to fulfil these requirements, such as lipid NPs, placing them in the top of the FDA-approved nanomedicines, including the COVID-19 vaccine formulations. However, most of them are unstable and prone to degradation in the harsh gastric environment, leading to premature release of their cargo or their limited storage stability [17,176].

Regarding inorganic materials, silica is one of the most promising materials for the construction of nanovaccination and delivery systems. MSNs have high loading capacity, due to their large specific surface area, and present good performance in delivery and controlled release, due to the tunable hollow and mesoporous structure. In addition, they can be degraded, thus allowing their excretion through the urine, which is of interest for their use in vaccines. Recently, Hong et al. reported the synthesis of 80 nm and negatively charged MSNs able to drain to lymph nodes and demonstrated that their ability of antigen cross-presentation can be tuned by adjusting their pore size [177]. In addition, they showed that large-pore MSNs loaded with B16F10 tumor antigens presented an antitumor effect in vivo, without additional adjuvants. Some other studies revealed that SBA-15 nanoparticles displayed increased phagocyte intake and low contact with the cells possessing greater immunogenicity and immunological response than Al(OH)_3_ [178]. Furthermore, the highest concentration of SBA-15 also led to a significant increase in the number of cells, creating interleukin (IL)-4 and interleukin (IL)-13 and generating a heterogeneous reaction of both Th1-type and Th2-type cytokines. Mody et al. reported the synthesis of silica vesicles based on hollow structures as new generation antigen carriers and adjuvants for bovine viral diarrhea virus (BVDV) vaccines, which showed high loading capacity and controlled release as immunogenic determinants of BVDV, namely codon-optimized E2 protein. After in vitro screening, their silica vesicle formulation achieved 10 times higher antibody response, when compared to a conventional adjuvant in mice, as well as higher cell-mediated responses. Thus, silica vesicles can serve as new-generation adjuvants and nanocarriers for improved veterinary vaccine delivery [179].

*Mycobacterium tuberculosis* (Mtb) produce membrane-derived extracellular vesicles containing the proteins responsible for modulating the pathological immune response after infection. In order to mimic these extracellular vesicles, Montalvo-Quirós et al. developed MSNs that were externally functionalized with immunomodulatory proteins (Ag85B, LprG and LprA). By evaluating the amounts of pro-inflammatory (TNF) and anti-inflammatory (IL-10) cytokines in the exposed macrophages, the immunostimulatory potential of the proposed nanosystems was proven [180]. With the increasing necessity to develop vaccines against COVID-19, MSNs have been tested as interesting vaccines platforms. Biodegradable MSNs were used to encapsulate epitope peptides, showing excellent safety and epitope delivery, inducing a robust peptide-specific immune response in mice [181].

MSNs were also used to prepare vaccines against *Schistosoma mansoni* by loading them with soluble worm antigenic preparation (SWAP) coming from adult *Schitosoma*. SWAP-loaded MSNs, which showed higher immunization performance, compared to a conventional immunization system (SWAP-associated aluminum salt), suggesting that they are a promising strategy for improving the immune response against *Schistosoma mansoni* [175]. All the published studies around silica nanoparticles as adjuvants highlight their importance in the future of the vaccine development.

#### 3.1.4. Other Biomedical Applications

Tissue engineering is another biomedical field where the MSNs have been applied. They could improve the effectiveness of bone and vascular tissue regeneration and wound healing, being a simultaneous reservoir of bioactive factors and scaffold to mimic the natural extracellular matrix [47], or even stimulate immunomodulation [182] or cell proliferation molecular pathways [183], as has been proved in murine models. Mesoporous bioactive glasses (MBGs) were reported by Hench et al. [184] as a new class of biocompatible materials that show both osteoconductive and osteoproductive behaviors, as well as an enhanced bone regeneration and proliferation [185]. MBGs play an important role in bone regeneration because of their striking textural properties, quick bioactive response and biocompatibility. As other bioactive glasses, MBGs are mainly formed by silicon, calcium and phosphorus oxides, whose ions play a key role in cell proliferation, as well as in homeostasis and bone remodelling process. MBGs present noticeable textural properties, biocompatibility and quick bioactive response. For regenerative medicine (RM), it is common to add to the bioactive glasses small amounts of oxides of specific elements conferring them additional biological capacities, such as osteogenic, angiogenic, antibacterial, anti-inflammatory, hemostatic, or anticancer properties [186]. Three-dimensional scaffolds and nanoparticles based on CaO–P_2_O_5_–SiO_2_ MBGs decorated with stem cells have been reported as a promising approach for RM of bone tissues [114,115].

MSNs have also been increasingly employed as an ideal candidate for stem cell therapies, contributing to stem cell maintenance and differentiation by altering the properties of the scaffold or providing the signals required for stem cell function [187,188]. In addition, they can be used for photodynamic therapy, radiation therapy, or immunotherapy, among others by combining MSNs platforms with specific cargos, such as photosensitizers or radioisotopes [47]. J. Mosquera et al. reported a new method for enhancing the biological stability of Au NPs by coating them with silica shells, as an alternative for protecting the gold core from the direct attachment of biomolecules (protein corona) and avoiding undesired effects [189].

Likewise, plasma complex component functionalized manganese-doped silica nanoparticles with a redox response have been designed as a targeted drug carrier for resveratrol, which effectively transports insoluble drugs to cross the blood–spinal cord barrier. Manganese-doped silica nanoparticles-resveratrol may be potentially useful for the treatment of spinal cord injury by reducing neuronal apoptosis and inhibiting inflammation caused by reducing oxidative stress to promote the recovery of mouse motor function [190].

## 4. Coating and Functionalization

MSNs present a lot of advantageous properties, with the facility of functionalization being highly relevant, as it allows their functionalization with a variety of moieties in the different regions of the particle. MSNs have a portion of the silicon atoms in the tetrahedral structure linked to hydroxyl groups (OH-, silanol sites). Due to the presence of such a large number of connectivity defects, functional groups can be introduced, making MSNs extremely versatile and potentially adaptable for their use in specialized tasks [64]. Positively charged drugs that are soluble in water prefer to be adsorbed onto the negative charged pores and surfaces of MSNs, forming stable MSNs/drug complexes [191]. On the other hand, hydrophobic drugs are typically incorporated into the pores and surface of MSNs via hydrophobic interactions and weak intermolecular bonds. Hydrogen bonds result in weak interactions, allowing the drugs to be contained and released in a sustained manner in the desired location. In some cases, the drug is released easily and prematurely before it reaches the desired site. As a result, on several occasions, NPs must incorporate surface modifications, such as coatings, to prevent the drug from being released in inappropriate locations or in an uncontrolled manner [192]. MSNs are generally hydrophilic, due to the silanol groups present on their surface, but they can be rendered hydrophobic easily by functionalizing their surfaces with various organic groups [7], either on the silicate surface, inside the silicate walls, or by trapping within the channels [25]. The ease with which siloxane (Si-O-Si) and silanol (Si-OH) groups on the surface of the material can be modified is critical for the functionalization and drug adsorption processes. While the possibility of selecting the pore size opens up a variety of possibilities for hosting different molecules, references dealing with the inclusion chemistry of MSNs are scarce and typically involve structural modification. The structure of the wall of the pores may also act as reacting nuclei for specific guest chemical species [59]. Thus, Si can be functionalized similarly to control the drug’s diffusion kinetics. The functionalization of its surface confers specific abilities on the target site, in addition to improving the drug’s blood circulation time and immune response. The systemic delivery of toxic biomolecules, particularly chemotherapeutics, via MSNs-based nanocarriers may have unintended cytotoxic effects on healthy cells. To minimize this systemic cytotoxic effect, MSNs can be functionalized with specific cell targeting ligands that enable the nanocarriers to be localized to the designated cells/tissues via cell-specific receptors [40].

MSNs can be modified by co-condensation or post-synthetic grafting. Co-condensation of hydrolyzed alkoxysilanes and organoalkoxysilanes result in a directed modification of the inner pore surface with high functional group loading (previously mentioned MONs). As silica is condensed, the organoalkoxysilane is co-condensed, positioning the organic moiety directly onto the pore walls. Its primary disadvantage is the functional group deposition on the inner channel walls, as well as the outer surfaces of the MSNs, in a non-specific manner. Consequently, the final materials exhibit an inhomogeneous distribution of the functional groups, with higher density around the pore mouths and the external surfaces of the MSNs. The second technique, called post-synthetic grafting, involves the modification of the surface of MSNs after they have been synthesized. This method employs the surface-accessible silanol groups both within the mesopore network and on the outer surface [25,193]. Post-synthetic grafting has several advantages, including the retention of a significant amount of the MSNs’ mesostructure and mesoporosity, following the introduction of functional groups. Additionally, the organic groups can be easily grafted onto the available surface of the MSNs. In comparison to stepwise post-synthesis grafting, the co-condensation approach allows for the modification of the surface of MSNs with organic functional groups in a single pot. Through covalent or electrostatic interactions, it is possible to introduce a diverse array of organic molecules to the silanol groups on the external surface of the MSNs [70].

### 4.1. Active and Passive Targeting

The surface functionalization of MSNs is being highly studied, as it can allow us to enhance the drug delivery specificity and to avoid possible side effects. MSNs are versatile carriers, as they can be loaded with large variety of drugs with different physicochemical properties and can be functionalized for effective therapy. Active targeting improves the uptake of nanoparticles into cells by attaching targeting molecules (ligands) to the particle surface. Based on the difference between healthy and diseased cells, suitable receptors that are usually overexpressed on the diseased tissues or tumor endothelium are selected, and ligands specific to those receptors are conjugated on the surface of the MSNs, allowing endocytosis-mediated entry into the target cells [1]. The efficiency of recognition and therefore, the internalization, depends on the abundance of receptor, the density of ligand anchored to the nanoparticle and the affinity between both. Among the wide variety of strategies most commonly used for ligand binding on the surface of MSNs are amide formation, disulfide bridges and click chemistry [194]. Conventional targeting labels include small molecules, aptamers, saccharides, antibodies, vitamins, proteins and peptides among others [195].

On the other hand, passive targeting can be described as the deposition of drug or drug carrier systems at a specific location, due to pharmacological or physicochemical factors, and it can be achieved by exploiting pathological conditions in diseased tissues. For example, chemotactic factors released in inflamed tissues can produce an increment of the vascular tissues permeability, decrement of the pH and increment of the temperature being useful for passive targeting [196,197]. The enhanced permeability and retention (EPR) effect, especially studied in cancer, has enabled the passive targeting of nanocarriers. This effect relies on the tendency of macromolecules and particles of certain sizes, such as nanoparticles, to preferentially accumulate in tumor tissues. Tumor vasculature is typically more permeable and lacks effective lymphatic drainage. Generally, nanoparticles with sizes ranging from 30 nm to a few hundred nanometers can pass (by passive accumulation) to tumor sites using the EPR effect [198]. As a result of this and the low lymphatic drainage, the NPs may penetrate and accumulate in angiogenic tumor areas [199]. However, the EPR effect is not universal for all types of tumor cells and a lack of cell-specific interactions might decrease the therapeutic efficacy and induce multiple drug resistance [169].

The incorporation of biomolecules on the external surface of the silica nanoparticles can serve both to target the site of interest and to escape the phagocytosis of cells [200] and provide a protective effect, thus delaying the action of the reticuloendothelial system (RES) [201].

### 4.2. Functionalization in Drug Delivery

**Figure 7:**
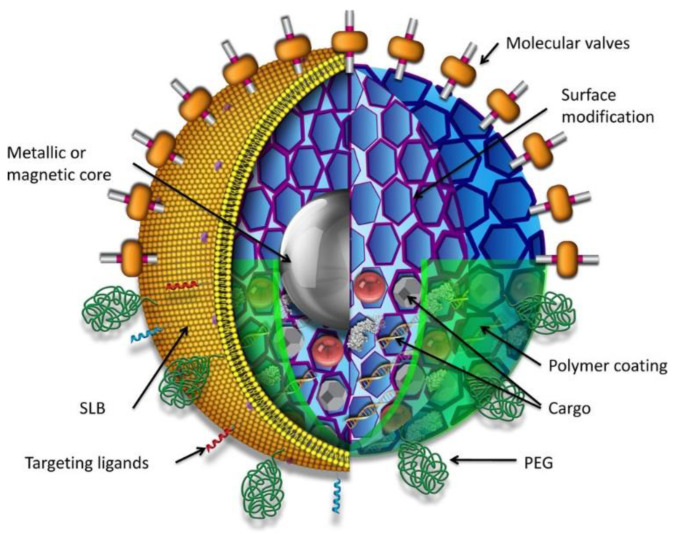
Schematic representation of a multifunctional mesoporous silica nanoparticle showing possible core/shell designs, surface modifications and multiple types of cargoes [193]. Copyright © 2022, American Chemical Society.

MSNs undergo surface modifications through either covalent chemical reactions or non-covalent interactions. Functional groups can be attached onto the surface of MSNs to control the rate and location of drug release and to reduce the toxicity of the MSNs [23]. Thus, MSNs for sustained drug delivery can be categorized into two groups, namely unmodified and modified silica materials. By adjusting the pore structure and diameter and the size of the nanocarriers, the use of unmodified silica for sustained drug release can be achieved. In the case of modified silica materials conjugated to organosilanes, the drug dissolution from MSNs can be delayed by the interaction between the drug molecules and the functional groups, allowing for a more sustained release. In addition, active targeting for the specific drug delivery can be achieved with the decoration of MSNs with targeting ligands such as antibodies and peptides. Moreover, the conjugation of magnetic materials onto MSNs allows for their use as homing devices (Figure 7). The particle size and surface modification of MSNs have a significant effect on the pharmacokinetics and biodistribution of the particles during the targeting process. These parameters have a direct effect on particle stability, circulation time, tumor accumulation, cellular uptake and therapeutic efficacy, so they should be taken into account when aiming for high targeting efficiency [106].

SiO_2_ NPs can be functionalized with organic macromolecules, such as dendrimers, phospholipids and polymers, to increase their water solubility, biocompatibility and resistance to unwanted reactions between the biological environment and nanocarriers. Additionally, these modifications have an effect on their biodistribution and accumulation in the body [202]. It is well-established that the physicochemical properties of the nanoparticles play a significant role in their cell internalization, with their surface charge being a critical factor. With a large internal and external surface, the surface potential of MSNs can be precisely controlled by the functionalization with charged chemical groups [83]. The presence of silane groups in silica can induce a greater affinity toward phospholipids, which favors their capture by the cells of our body. Due to the presence of Si-O bonds, silica nanoparticles are generally considered stable against external stimuli, such as mechanical stress and degradation, thereby eliminating the need for the stabilizing bonds used in other release systems [203]. However, the bioactive molecules that are physically trapped in the MSNs could be uncontrollably released with an initial burst. One problem that arises with the presence of silanol groups on the surface of MSNs is that they could inhibit the bioactivity of proteases or cause hemolysis, due to the interaction with phospholipid layers on the membrane of red blood cells. However, to improve the properties and expand the potential applications of MSNs, these silanol groups can be easily modified with other functional ligands [40]. Common coatings for MSNs include different polymers, such as polyethylene glycol (PEG), polyethyleneimine (PEI)-PEG copolymer, or poly (N-isopropylacrylamide). The incorporation of organic groups into mesoporous materials enables the surface properties (hydrophilicity, hydrophobicity, acidity, basicity and binding to guest molecules) to be tuned, in addition to altering the surface reactivity and protecting the surface from chemical attacks [25]. Modification of the pore walls by coating the mesoporous structure can also alter the adsorption behavior of the material [204]. MSNs must remain highly dispersed, in order to be used in biomedical applications, which requires colloidal stability. When MSNs are aggregated, internalization is impaired, biodistribution becomes difficult to control and larger effective particle sizes may result in increased toxicity. Chemical modification of the surfaces, incorporation of protein or polymer coatings, and coating with a supported lipid bilayer can all help to reduce particle agglomeration [193]. Additionally, the surface modification of bare MSNs can significantly reduce, or even eliminate, thrombogenic effects and nonspecific protein adsorption on the surface of MSNs [169].

Therefore, MSNs can be modified with a large range of functional moieties to allow for a better targeting of the desired area. The aforementioned tables (Table 1 and Table 2) present multiple functionalization strategies of MSNs to be applied in the biomedical field. As it can be seen, several recent studies introduce specific functionalization to the MSNs to allow for active targeting to specific sites, for example, vascular endothelial growth factor receptor, VEGFR for antiangiogenic therapy [135], folic acid for breast cancer [148], or PSA in prostate cancer [160].

Smart drug delivery capability can be added to MSNs by new mechanisms, known as nanogates, which block the output of the therapeutic agent until its release is activated through certain stimuli, thus avoiding undesired release and side effects [43,108,205].

#### 4.2.1. PEG Functionalization

**Figure 8:**
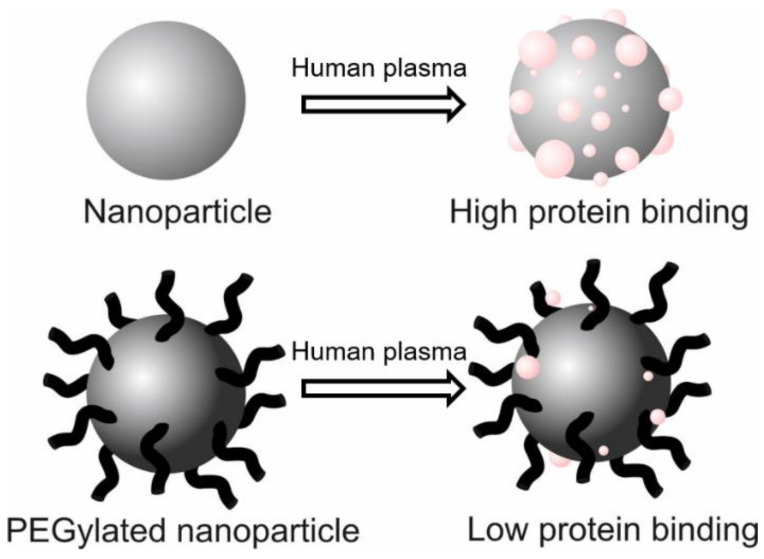
Schematic representation of the interaction of NPs with human plasma. PEGylated NPs are able to prevent the adsorption of proteins from the blood, increasing circulation time. Adapted from [208]. Copyright © 2022 Elsevier B.V. All rights reserved.

PEG and its derivatives have been used to modify the surface of particles in controlled-release systems, such as liposomes, emulsions, microspheres and sensor technology, in recent years, due to their low cost, versatility and approval by the food and drug administration (FDA) for a variety of commercial applications [206,207]. PEG is a spiral polymer composed of repeating units of ethylene ether with dynamic conformations. It has the ability to resist the adsorption of blood proteins (Figure 8), as well as induce a reduction in platelet aggregation, neutrophil activation, hemolytic activity and coagulation, due to its hydrophilicity and coordination with water molecules in aqueous media [208,209]. Studies have shown that surfaces functionalized with PEG are biocompatible, hydrophilic, resistant to adhesion and biological degradation, and able to prevent the nonspecific adsorption of proteins. PEG also increases the circulation time of the functionalized materials by reducing the rate of clearance through the organs, such as the kidney. Thanks to its nontoxic and non-immunogenic properties, it has been used to produce biocompatible surface coatings on silica films and nanoparticles. PEG is also useful to prevent the aggregation of particles, as it can work as a steric stabilizer in aqueous solutions [112]. The coagulation rate of NPs can be tuned within a broad range by modifying the chain length and the degree of coverage of PEG on the particle surface [205].

All of these characteristics increase NPs’ blood circulation time and biostability for in vivo applications [210]. Mean circulation time (t_1/2_) describes the residence of accumulation in the blood and the period during which the concentration of circulating NPs remains above 50% of the injected dose, which is analogous to the half-life of a drug. For NPs to be effective, they must have a long enough half-life to reach the target and must also remain in the affected area long enough for imaging or drug administration [209]. PEGylated NPs exhibit a variety of properties, as a result of steric impediment and repulsion. Steric repulsion toward macrophages and blood proteins is affected by the different conformations and molecular weights of PEG chains as they modify their flexibility and hydrophilicity [211]. There are several forms of PEGylation, depending on the employed species and its molecular weight, including maleimide PEG (PEG-Mal) [212], PEG-silane [94,95], 2-[methoxy(polyethyleneoxy)6-9propyl]trimethoxysilane (MPEGTMS) [200], methoxy-PEG-pyridyl disulfide (mPEG-SS-Pyridine) [201], methoxy-PEG-Carboxyl (mPEG-COOH) [213], diacid PEG (COOH-PEG-COOH) [214], methoxy-PEG-Amine (mPEG-NH_2_) [215], and polyethylene glycol methyl ether (PEGME) [206]. He et al. [200] demonstrated that the clearance time and subsequent organ accumulation depend on the surface modifications of the particles (-OH, -COOH and -PEG), with the functionalization of the PEG chains probably being the most efficient way to avoid nonspecific accumulation [216]. As previously mentioned, the binding of PEG on the surface induces a change in the behavior of the NPs. PEG-coated NPs increase their t_½_ by reducing the opsonization process, thus avoiding recognition by monocytes and macrophages and allowing the NPs to remain in the blood. Moreover, polyethylene glycol modifies flexibility and hydrophobicity and increases “softness”, with respect to the underlying material, thereby affecting extravasation [209]. To design effective MSNs for diagnosis or therapeutic application, all the steps in a given biological process need to be considered, as a particular surface chemistry, surface charge, or surface functional group may favor one of the steps in the given biological process, but inhibit others. Therefore, MSNs must be designed in such a way that they are capable of crossing cell membranes, evading endosome capture, avoiding unwanted intracellular binding processes, and finally reaching their cellular targets and releasing their payloads of drugs, biomolecules and bioactive agents, as required [7].

#### 4.2.2. Smart Drug Delivery

The creation of mechanisms for on-demand release of drugs contained in MSNs has been highly studied. These mechanisms, known as nanogates, can allow for the controlled release of the drug only when they are exposed to certain stimuli. These stimuli can be either internal (pH, redox potential, temperature, biomolecules), external (light, magnetic field), or even the combination of both (dual-stimuli or multi-responsive release systems) [43,108,205], with the pH and redox stimulus being the most commonly used simultaneously [217].

For example, the pH of the tumor microenvironment is more acidic (about 6.5−6.8) than healthy one (about 7.0–7.4), and this can induce the cleavage of acid-sensitive molecules [218]. Gatekeepers sensitive to overexpressed glutathione (GSH) are also interesting. In blood and extracellular matrices, the GSH levels are low (2–20 µM), while in cells, the levels are higher (0.5–10 mM). The pore entrances would remain sealed in the bloodstream, where the low GSH levels found there are unable to break the bonds, but when MSNs are internalized, the high production of GSH in the cytosol would cleave the bonds and open the pores, releasing the drugs. This can minimize the presence of free drugs in the bloodstream [219].

**Figure 9:**
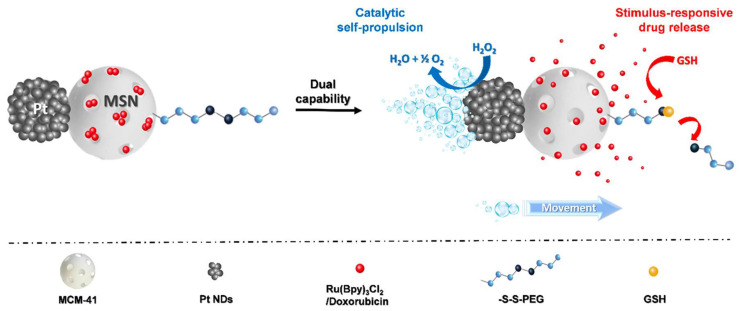
Schematic representation of Pt-MSNs nanomotors with a catalytic self-propulsion and a glutathione-mediated [222]. Copyright © 2022 American Chemical Society.

There are essentially three routes that can be used to achieve on-demand release in MSNs: use responsive polymer coating to modify the surface of MSNs, attach ligands to the mesopores (gatekeepers), or anchor anticancer agents to the MSNs with responsive cleavable linkers [220]. “Gate keepers” are used to seal MSN pores and are only opened under exposure to specific stimulus, thus avoiding adverse effects and improving the anti-cancer effects. They include inorganic materials, polymers, biomacromolecules, drug molecules themselves and biological membranes. There are numerous strategies for modifying the surface of MSNs with functional groups and ligands to improve their physicochemical properties, including sensitivity to temperature, pH, light, magnetic fields, electric fields, redox agents, ultrasound (US), glucose and enzymes, in addition to site-targeted ability, improvements in biocompatibility, biodegradability, excretion and the ability to control the release of loaded bioactive molecules [40]. These novel developments position surface-functionalized MSNs as ideal candidates for intracellular controlled-release delivery [221]. In this field, bioinspired nanomachines or self-propelled nanomotors for the intelligent controlled release of therapeutic drugs have attracted much attention in the last years. Innovative multifunctional gated platinum−mesoporous silica nanomotors have been designed by Martínez-Máñez et al. [222]. These nanomotors have an ultrafast self-propelled motion, caused by the catalytic decomposition of low concentrations of hydrogen peroxide. This fast and directional displacement, and the reduction of the disulfide bonds of the capping ensemble by intracellular glutathione levels, facilitates the rapid cellular internalization, as well as the on-demand specific release of a cytotoxic drug into the cytosol (Figure 9). Table 1 presents several recent examples of studies that functionalize MSNs to allow for the on-demand release of drugs. 

#### 4.2.3. Other Coatings and Functionalizations

Other surface modifications of MSNs have also been explored. To regulate the in vitro cellular uptake and cytotoxicity, as well as the in vivo biodistribution and excretion of MSNs, different surface functionalizations have been suggested, including amino, phenyl, carboxyl and methyl phosphonate groups with positive, neutral and negative zeta potentials, respectively [18]. For example, by modifying the surface of MSNs with positively charged groups, which have high ROS-associated toxicity, enhanced passive bioaccumulation via the EPR effect could be achieved because of nonspecific interactions between the positive charge NPs and negative charge of the cell membrane (and thus, more permeability), which plays an important role in endocytosis of NPs in passive targeting to neoplasm cells [223,224]. Additionally, MSNs can be coated with a variety of compounds, thus expanding the range of functional groups that can be anchored to the surface of the NPs, including epoxides or cyano groups, to name some [225]. For example, the MSN coating with polycations, such as poly(L-lysine), poly(ethyleneimine) (PEI), polyamidoamine dendrimers and natural chitosan, and an endosome-disrupting peptide have allowed for the enhancement of their cellular uptake and endosomal release [7].

## 5. Biocompatibility, Degradability and Biodistribution

The safety and toxicity of nanoparticles are a major concern, owing to their high surface-to-volume ratio. Biocompatibility is a necessary attribute for ensuring that these products do not accumulate indiscriminately in the body over time, resulting in adverse effects [1]. To avoid the accumulation of nanoparticles within an organism, they must be either excreted or biodegraded in biological systems. Approved pharmaceutical products must not accumulate in the human body, in order to avoid severe and unpredictable side effects. The degradation, biodistribution and biocompatibility of SiO_2_ NPs will largely depend on the medium but can be tuned by adjusting several parameters, including porosity, size, shape, surface chemistry and dispersibility (largely depending on the hydrophilicity) of the particles [226]. The role of shape and morphology of the MSNs on their interaction with living cells has been investigated. Lin et al. [227] studied the cellular uptake efficiency of tubular and spherical MSNs in cancerous and non-cancerous cell lines. They observed that the efficiency and rate of internalization were exclusively dependent upon morphology and particle aggregation [63]. There are contradictory studies about this topic and different opinions reporting that spherical particles show advantages over their rod counterparts during cellular uptake [228]. Recently, other researchers provided additional evidence for the critical role of particle morphology in particle-cell interactions [64]. In contrast to other chemo-physical parameters, the effects of surface properties have received significant attention from the scientific community.

Despite the great interest that MSNs hold as DDS, they have not yet been approved to be used in the clinic by the regulatory agencies. MSNs degradation, biodistribution, and clearance routes are relevant prerequisites that need to be known before reaching clinical trials. Therefore, the stability of the nanoparticles in the blood or physiological media is one of the key parameters that needs to be determined [17,81].

### 5.1. Biocompatibility and Toxicity

The primary mechanism of silica toxicity is through its surface chemistry (silanol groups), which can interact with the membrane components, resulting in cell lysis and leaking of the cellular components [1]. Some studies have shown that nanoparticles with large surface areas and abundant silanol groups produce reactive oxygen species (ROS), cytokines and chemokines, which induce inflammatory responses and play a significant role in nanomaterial-induced injuries [15,18]. Surface silanol (-Si-OH) groups can either link via hydrogen bonds to membrane components or dissociate to form SiO^−^ (above the isoelectric point of silica, see Figure 6) and interact electrostatically with phospholipids that are positively charged, which result in intense interactions and possible membranolysis. Indeed, solid NPs demonstrated increased hemolytic activity (cytotoxic effects), when compared to MSNs, due to the fact that MSNs have a large surface area, but most of these silanol groups are hidden inside the pores. This is the reason why MSNs present higher biocompatibility with red blood cells [13]. Generally, the toxicity of NPs increases in a dose-dependent manner. Napierska et al. [229] concluded that the size of the nonporous silica nanoparticles highly affect their cytotoxicity. Particles with small size have a greater surface area per mass, so more silanol groups are on their surface, which can be in contact with the cells and thus exhibit higher toxicity than larger particles.

The importance of silanol groups is further demonstrated by the fact that treating the silica surface to decrease the number of silanol groups can reduce, or even switch off, the hemolysis of red blood cells [193]. The interaction between the negatively charged silica and positively charged trimethylammonium groups of lipid bilayer could be reduced with the pegylation of the NPs, improving their hemocompatibility [89]. In addition, by altering the surface using PEG, MSNs are able to avoid quick accumulation in the liver, spleen, and lung tissues. This is because, as already mentioned, PEG-MSNs have a prolonged circulation time. The findings reported by Yu et al. [230] suggested that the toxicity depends on the type of cells and the NPs’ concentration, pore size, surface charge and functionalization that, in turn, affects their biodistribution [1]. As a result, it is critical to determine whether the NPs or their constituent materials may result in toxicity in the blood and/or vital organs [216].

Studies showed that ethenylene-bridged PMO colloids were very low hemolytic materials, in comparison with MSNs and non-porous silica NPs, showing the importance of testing the biological properties of each newly synthetized nanoparticle [93].

A second factor that may contribute to toxicity is the formation of ROS by the reaction of radicals on the silica surface and water, i.e., the outstanding hydroxyl radical, which is one of the most reactive species in nature. ROS can cause cell death by necrosis, with the disruption of cell membranes, or by apoptosis (programmed cell death). Mutagenesis and carcinogenesis can also be promoted in sublethal doses, as ROS can upregulate the production of cytokines and other inflammatory mediators. Although numerous recent studies have been conducted, it is generally observed that MSNs are significantly less toxic than their nonporous colloidal silica counterparts, presumably because the mesoporosity (presence of voids) reduces the effective MSNs/membrane contact area [193].

When NPs encounter the body, blood proteins aggregate. This phenomenon is referred to as protein corona. Protein corona is a dynamic coating of proteins that binds to the outer surface of the MSNs, concealing them as they travel through the body. This protein corona is formed when the proteins adsorb on the MSN surface and create a shell around them. The nature of the proteins that conforms to the corona is important, as they can change the interaction of the MSNs with the body, for example, by modifying the stability, charge, size and surface chemistry of MSNs, thus having an impact on its cellular uptake and toxicity. Effectively, the presence of the protein corona reduces the hemolytic effect induced by the surface of the MSNs, thereby affecting cellular internalization. Additionally, the protein corona may facilitate particle passage through the blood–brain barrier or uptake by monocytes or macrophages [23]. Nonetheless, nearly all types of proteins found in the media (95%) are adsorbed onto MSNs in a variety of configurations. This is consistent with the fact that the protein corona is predominantly dynamic, rather than static. Additionally, the protein adsorption occurs via two mechanisms, including the reversible adsorption of loosely bound proteins (soft corona) and the irreversible adsorption of strongly bound proteins (hard corona) [89]. Strategies to reduce protein binding have been discussed in the previous section.

Regarding cellular biocompatibility and cytotoxicity, many in vitro and in vivo studies have been performed, in which MSNs can be internalized into the normal and cancerous cells, without affecting the growth of the cells, their proliferation, and cellular differentiation [231].

### 5.2. Degradability

The degradability of MSNs is a critical point to assess, especially for their application in the biomedical field. It is a current controversial topic and an ongoing discussion in research circles [89,226].

**Figure 10:**
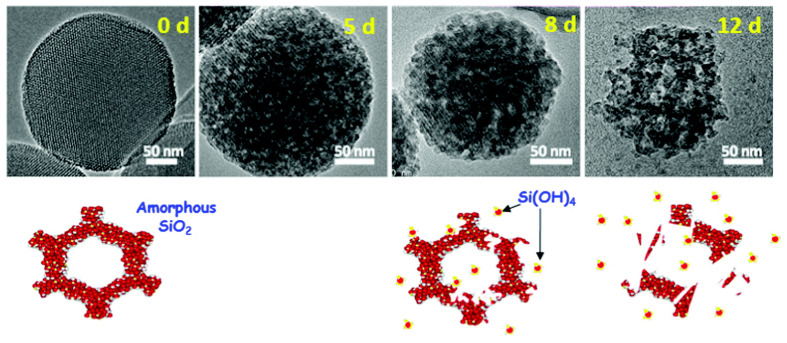
TEM images showing MSN degradation in PBS after 0, 5, 8 and 12 days, and schematic representation of their dissolution process in an aqueous medium. Reproduced from [17]. Under CC BY 3.0 license (https://creativecommons.org/licenses/by/3.0/, accessed on 27 November 2022).

When MSNs come in contact with the inside of the body, hydrolysis reactions take place on their surface that break apart the silica nanoparticles into silicic acid, preventing their toxic buildup. Particles delivered to the body are easily degraded via a three-step reaction mechanism. Firstly, water adsorbs onto the surface of MSNs and then, a hydrolysis reaction happens between water and the siloxane groups converting them into silanol groups, which take part in an ion-exchange reaction. This reaction causes the Si to leave the nanostructure in the form of silicic acid, which is considered nontoxic and can be eliminated from the body with the urine [23,90]. Therefore, in terms of biodegradation, the orthosilic acid by-products are not expected to be cytotoxic, since they are biocompatible and excreted through the urine. This is why silica has been “generally recognized as safe” by the US Food and Drug Administration (FDA) for over 50 years, which accounts for its current use as a food additive in various commercial products [89]. The dissolution rate of silica is determined by both the particle characteristics (surface area, pore size, pore-wall thickness, condensation degree, aggregation state, surface functional groups, concentration, etc.) and the properties of the degradation medium (pH, temperature, concentration, static or continuous flow, etc.) [1,89,226]. Moreover, surface functionalization can help to prevent MSNs from degrading rapidly and prolong their stability in biological media. Figure 10 shows an electron microscopy analysis of SBA-15 bearing different surface functionalities after being exposed to phosphate-buffered saline (PBS). SBA-15 without functionalization suffers a total loss of mesostructural order, whereas, when it is functionalized with methyl groups, it shows small ordered domains of 2D-hexagonal structure. If SBA-15 is functionalized with octyl chains or aminopropyl groups, it shows a well-ordered 2D-hexagonal structure, which is preserved after the long-term assay. There are also other strategies to improve the degradability of the MSNs. For instance, Guo et al. [232] doped MSNs with strontium ions and showed that Sr-doping significantly improved the in vitro degradability and cytocompatibility of MSNs in a Sr content-dependent manner. So, inorganic moieties incorporation into MSNs can also tune the dissolution rate of MSNs. Zirconium doping was also shown to virtually stop the degradation of the particles, while calcium, manganese, or iron presented the opposite effect. Not only the inorganic doping, but also the presence of specific proteins or pH, affected the degradation kinetics. For example, silica-iron oxide nanocomposites showed higher degradation in fetal bovine serum than in water for the same period of time [233]. These particles presented a unique cavity-like mesostructure with large pores containing iron oxide crystalline nanophases with a homogenous distribution of silica-amine and iron oxide.

By incorporating organic moieties into silica NPs, the degradation of silica can be also be tuned [226]. The noncovalent silica doping with organic molecules as photosensitizers accelerates their dissolution. In contrast, the aforementioned MONs and PMO NPs with covalent organically bridged silsesquioxanes presented much lower dissolution rates than MSNs, which was attributed to the high hydrophobicity and the more stable Si–O-bonds in silsesquioxanes [17,226]. Ethylene-, ethenylene- and phenylene-based PMO that present lower dissolution rates can be useful for long-term bioimaging and sustained-drug-delivery applications. However, faster particle degradations in biorelevant conditions are needed for many biomedical applications, so this higher stability can be modified by incorporating organo-bridged alkoxysilanes in their framework, which can be cleaved enzymatically or via redox reactions, among others. Diverse studies have been performed with PMO NPs containing redox-senstitive bonds (e.g., disulfide bonds), with their degradation being faster than the MSNs without redox-sensitive ones [90,94]. Having a controllable biodegradation is of interest, since it will determine the in vivo fate of the nanoparticles.

### 5.3. Biodistribution

Biodistribution is the study of the static and dynamic distribution of a given compound within a biological system or within an organism. The biodistribution of the MSNs, once they enter into the body, will depend on their physicochemical characteristics and their ability to avoid certain obstacles, which will, in turn, also determine their toxicity [202]. Additionally, the uptake mechanisms depend on the surface functionalization and the resulting surface charge, size and shape [15].

Controlling for particle size and homogeneity is critical in biodistribution, as they vary nonlinearly between organs. Regulating the hydrodynamic diameter and increasing the colloidal stability of the MSNs is also critical because aggregated particles tend to behave similarly to large-sized MSNs, despite their small size [30]. Furthermore, it is necessary to determine the most appropriate route of administration, as this will also determine its biodistribution [234]. Circulating NPs must overcome a number of physiological and biological barriers, including degradation, capture by macrophages in the RES, formation of the protein corona, accumulation in undesirable locations, etc., which might hinder the transport of the payload they contain, from the point of administration to the target area [235]. For example, hydrophobic nanoparticles are generally rapidly cleared from the circulation by cellular elements of the RES, particularly in the liver and spleen, having short half-lives (seconds or minutes) in preclinical models [198]. The geometry of the particles (size and shape) plays an essential role in the speed and the internalization mechanism of target compounds. For example, particles <8 nm in size are rapidly eliminated from the body by renal filtration [9]. Conversely, the liver and spleen entrap particles of >200 nm, while the liver ensures the elimination of NPs of <200 nm [198]. Additionally, the shape of silica NPs dramatically affects their toxicity (inflammatory responses, etc.) [236]. Materials with large surface areas and elongated shapes can create impediments that spherical materials do not [237]. These findings emphasize the critical role of shape control in promoting the circulation and tissue penetration properties of nanomaterials [135]. Often, the strategy entails functionalizing the surface of the NPs with ligands associated with overexpressed receptors in specific cells [10]. The surface functionalization plays a key role in the overall biodistribution. For instance, Lu et al. [238] reported that the majority of negatively charged MSNs are excreted through urine (major) 4 days after injection, indicating the rapid dissolution of MSNs in the body [83].

Thus, silica-based NPs are particularly appealing, due to their high biocompatibility, nontoxic degradation products and tunable hydrolytic degradability in biorelevant media, ranging from hours to days or weeks. Although few formulations based on silica nanoparticles are already in ongoing phase I and II clinical trials, MSNs nanocarriers for drug delivery are in the preclinical phase and have not been accepted by regulatory agencies for use in the clinic. Most of these preclinical studies have focused on small animal models, such as rodents, while only a few studies have been conducted in large animal models, including sheep, pigs and non-human primates [17].

## 6. Conclusions

The current review summarizes the recent advancements in the field of mesoporous silica-based nanoparticles. Due to their interesting properties, such as easily tunable morphological characteristics and high loading capacity, mesoporous silica nanocarriers have been frequently investigated as high-fidelity drug delivery systems. Consequently, MSNs exhibit significant potential as versatile and efficient nanocarriers, facilitating not only the transport and bioavailability of poorly soluble therapeutic or imaging agents, but also the delivery of highly toxic drugs, such as chemotherapeutic agents. Recent advances include the development of MSNs with site-specific properties that target and neutralize specific cells. MSNs with varying particle sizes, shapes and pore volumes can be obtained with relative ease, by merely varying the nature and concentration of the reagents, along with the conditions of the synthesis. Additionally, customizing the pore structure and the surface properties of the MSNs not only improves their loading capacities, but also allows for the modification of their drug-release profiles. Due to their ease of functionalization, different types of ligands can be attached to the surface of MSNs. The majority of research on MSNs is directed towards their use in cancer therapy, enabled by the targeted and controlled release of the cargo. Targeted MSNs are desired for molecular imaging, selective drug delivery, personalized therapy and the selection of candidate patients. Moreover, MSNs loaded with both therapeutic and imaging agents arise as promising theranostic approaches in multiple diseases or conditions. Regarding the translational evaluation of MSNs, in the last few years, research works have moved from synthesis and the in vitro characterization of potentially interesting MSNs to their functional characterization in biological systems. Both cellular experiments in vitro and in vivo experiments in animal models (mostly rodents) have been performed successfully to reliably support the potential biomedical applications of such MSNs, determining that MSNs cytotoxicity and biodistribution is dependent on their characteristics and the administration route. On the other hand, the implications associated with the long-term use of MSNs are currently being investigated to thoroughly demonstrate MSNs’ safety and biocompatibility in preclinical animal models. Nevertheless, given that phase I and II clinical trials evaluating silica nanoparticles are currently ongoing and have already shown encouraging results, MSNs are likely to move quickly into clinical trials for patients in most need of new therapies. In addition, already approved radioactive silica microspheres highlight the potential of these kinds of formulations to be applied for human use in the future. This is very encouraging and underscores the importance of continuing research in this field.

## Data Availability

Not applicable.

## References

[1] Narayan R., Nayak U.Y., Raichur A.M., Garg S. (2018). Mesoporous Silica Nanoparticles: A Comprehensive Review on Synthesis and Recent Advances. Pharmaceutics.

[2] Zhao P., Liu M.-C., Lin H.-C., Sun X.-Y., Li Y.-Y., Yan S.-Q. (2017). Synthesis and Drug Delivery Applications for Mesoporous Silica Nanoparticles. J. Med. Biotechnol..

[3] Deepak T., Michel D., Yashwant P. (2007). Nanoparticulate Drug-Delivery Systems: An Overview.

[4] Rahman I.A., Padavettan V. (2012). Synthesis of Silica Nanoparticles by Sol-Gel: Size-Dependent Properties, Surface Modification, and Applications in Silica-Polymer Nanocompositesa Review. J. Nanomater..

[5] Meier W. (1999). Nanostructure Synthesis Using Surfactants and Copolymers. Curr. Opin. Colloid Interface Sci..

[6] Fowler C.E., Khushalani D., Lebeau B., Mann S. (2001). Nanoscale Materials with Mesostructured Interiors. Adv. Mater..

[7] Asefa T., Tao Z. (2012). Biocompatibility of Mesoporous Silica Nanoparticles. Chem. Res. Toxicol..

[8] Jeelani P.G., Mulay P., Venkat R., Ramalingam C. (2020). Multifaceted Application of Silica Nanoparticles. A Review. Silicon.

[9] Heinz H., Pramanik C., Heinz O., Ding Y., Mishra R.K., Marchon D., Flatt R.J., Estrela-Lopis I., Llop J., Moya S. (2017). Nanoparticle Decoration with Surfactants: Molecular Interactions, Assembly, and Applications. Surf. Sci. Rep..

[10] Sun T., Zhang Y.S., Pang B., Hyun D.C., Yang M., Xia Y. (2014). Engineered Nanoparticles for Drug Delivery in Cancer Therapy. Angew. Chem. Int. Ed..

[11] Falgàs Comamala A. (2021). Antineoplastic Effect of Therapeutic Nanoparticles Targeted to CXCR4+ Diffuse Large B-Cell Lymphoma Cells. Ph.D. Thesis.

[12] Morales M.E., Castán H., Ortega E., Ruiz M.A. (2019). Silica Nanoparticles: Preparation, Characterization and Applications in Biomedicine. Pharm. Chem. J..

[13] Lin Y.S., Haynes C.L. (2010). Impacts of Mesoporous Silica Nanoparticle Size, Pore Ordering, and Pore Integrity on Hemolytic Activity. J. Am. Chem. Soc..

[14] Ibrahim I.A., Zikry A.A.F., Sharaf M.A. (2010). Preparation of Spherical Silica Nanoparticles: Stober Silica. J. Am. Sci..

[15] Graf C. (2018). Silica, Amorphous. Kirk-Othmer Encyclopedia of Chemical Technology.

[16] Zhang J.H., Zhan P., Wang Z.L., Zhang W.Y., Ming N.B. (2003). Preparation of Monodisperse Silica Particles with Controllable Size and Shape. J. Mater. Res..

[17] Vallet-Regí M., Schüth F., Lozano D., Colilla M., Manzano M. (2022). Engineering Mesoporous Silica Nanoparticles for Drug Delivery: Where Are We after Two Decades?. Chem. Soc. Rev..

[18] Tang F., Li L., Chen D. (2012). Mesoporous Silica Nanoparticles: Synthesis, Biocompatibility and Drug Delivery. Adv. Mater..

[19] Naik B., Ghosh N. (2009). A Review on Chemical Methodologies for Preparation of Mesoporous Silica and Alumina Based Materials. Recent Pat. Nanotechnol..

[20] Kumar S., Malik M.M., Purohit R. (2017). Synthesis Methods of Mesoporous Silica Materials. Mater. Today Proc..

[21] Kolbe G. (1956). Das Komplexechemische Verhalten Der Kieselsäure. Ph.D. Thesis.

[22] Stöber W., Fink A., Bohn E. (1968). Controlled Growth of Monodisperse Silica Spheres in the Micron Size Range. J. Colloid Interface Sci..

[23] Downing M.A., Jain P.K. (2019). Mesoporous Silica Nanoparticles: Synthesis, Properties, and Biomedical Applications.

[24] Díaz de Greñu B., de los Reyes R., Costero A.M., Amorós P., Ros-Lis J.V. (2020). Recent Progress of Microwave-Assisted Synthesis of Silica Materials. Nanomaterials.

[25] ALOthman Z.A. (2012). A Review: Fundamental Aspects of Silicate Mesoporous Materials. Materials.

[26] Buckley A.M., Greenblatt M. (1994). The Sol-Gel Preparation of Silica Gels. J. Chem. Educ..

[27] Li Z., Barnes J.C., Bosoy A., Stoddart J.F., Zink J.I. (2012). Mesoporous Silica Nanoparticles in Biomedical Applications. Chem. Soc. Rev..

[28] Lee M.H., Beyer F.L., Furst E.M. (2005). Synthesis of Monodisperse Fluorescent Core-Shell Silica Particles Using a Modified Stöber Method for Imaging Individual Particles in Dense Colloidal Suspensions. J. Colloid Interface Sci..

[29] Finnie K.S., Bartlett J.R., Barbe C.J.A., Kong L. (2007). Formation of Silica Nanoparticles in Microemulsions. Langmuir.

[30] Yamamoto E., Kuroda K. (2016). Colloidal Mesoporous Silica Nanoparticles. Bull. Chem. Soc. Jpn..

[31] Peres E.C., Slaviero J.C., Cunha A.M., Hosseini–Bandegharaei A., Dotto G.L. (2018). Microwave Synthesis of Silica Nanoparticles and Its Application for Methylene Blue Adsorption. J. Environ. Chem. Eng..

[32] Lovingood D.D., Owens J.R., Seeber M., Kornev K.G., Luzinov I. (2012). Controlled Microwave-Assisted Growth of Silica Nanoparticles under Acid Catalysis. ACS Appl. Mater. Interfaces.

[33] Pandey M.P., Kim C.S. (2011). Lignin Depolymerization and Conversion: A Review of Thermochemical Methods. Chem. Eng. Technol..

[34] Øye G., Sjöblom J., Stöcker M. (2001). Synthesis, Characterization and Potential Applications of New Materials in the Mesoporous Range. Adv. Colloid Interface Sci..

[35] Yanagisawa T., Shimizu T., Kuroda K., Kato C. (1990). The Preparation of Alkyltrimethylammonium-Kanemite Complexes and Their Conversion to Microporous Materials. Bull. Chem. Soc. Jpn..

[36] Zhao X.S., Lu G.Q., Millar G.J. (1996). Advances in Mesoporous Molecular Sieve MCM-41. Ind. Eng. Chem. Res..

[37] Schulz-Ekloff G., Rathouský J., Zukal A. (1999). Mesoporous Silica with Controlled Porous Structure and Regular Morphology. Int. J. Inorg. Mater..

[38] Grün M., Unger K.K., Matsumoto A., Tsutsumi K. (1999). Novel Pathways for the Preparation of Mesoporous MCM-41 Materials: Control of Porosity and Morphology. Microporous Mesoporous Mater..

[39] Trewyn B.G., Slowing I.I., Giri S., Chen H.-T., Lin V.S.-Y. (2007). Synthesis and Functionalization of a Mesoporous Silica Nanoparticle Based on the Sol–Gel Process and Applications in Controlled Release. Acc. Chem. Res..

[40] Hoang Thi T.T., Cao V.D., Nguyen T.N.Q., Hoang D.T., Ngo V.C., Nguyen D.H. (2019). Functionalized Mesoporous Silica Nanoparticles and Biomedical Applications. Mater. Sci. Eng. C.

[41] Slowing I.I., Trewyn B.G., Giri S., Lin V.S.Y. (2007). Mesoporous Silica Nanoparticles for Drug Delivery and Biosensing Applications. Adv. Funct. Mater..

[42] Wu K.C.W., Yamauchi Y. (2012). Controlling Physical Features of Mesoporous Silica Nanoparticles (MSNs) for Emerging Applications. J. Mater. Chem..

[43] Colilla M., González B., Vallet-Regí M. (2013). Mesoporous Silica Nanoparticles for the Design of Smart Delivery Nanodevices. Biomater. Sci..

[44] Grün M., Lauer I., Unger K.K. (1997). The Synthesis of Micrometer- and Submicrometer-Size Spheres of Ordered Mesoporous Oxide MCM-41. Adv. Mater..

[45] Le Y., Chen J.F., Wang J.X., Shao L., Wang W.C. (2004). A Novel Pathway for Synthesis of Silica Hollow Spheres with Mesostructured Walls. Mater. Lett..

[46] Wu S.H., Lin H.P. (2013). Synthesis of Mesoporous Silica Nanoparticles. Chem. Soc. Rev..

[47] Rastegari E., Hsiao Y.-J., Lai W.-Y., Lai Y.-H., Yang T.-C., Chen S.-J., Huang P.-I., Chiou S.-H., Mou C.-Y., Chien Y. (2021). An Update on Mesoporous Silica Nanoparticle Applications in Nanomedicine. Pharmaceutics.

[48] Yang P., Gai S., Lin J. (2012). Functionalized Mesoporous Silica Materials for Controlled Drug Delivery. Chem. Soc. Rev..

[49] Zhou W. (2000). HRTEM Investigation of Mesoporous Molecular Sieves. Micron.

[50] Beck J.S., Vartuli J.C., Roth W.J., Leonowicz M.E., Kresge C.T., Schmitt K.D., Chu C.T.W., Olson D.H., Sheppard E.W., McCullen S.B. (1992). A New Family of Mesoporous Molecular Sieves Prepared with Liquid Crystal Templates. J. Am. Chem. Soc..

[51] Anderson M.T., Martin J.E., Odinek J.G., Newcomer P.P. (1998). Surfactant-Templated Silica Mesophases Formed in Water: Cosolvent Mixtures. Chem. Mater..

[52] Zana R., Frasch J., Soulard M., Lebeau B., Patarin J. (1999). Fluorescence Probing Investigations of the Mechanism of Formation of Organized Mesoporous Silica. Langmuir.

[53] Cai Q., Luo Z.S., Pang W.Q., Fan Y.W., Chen X.H., Cui F.Z. (2001). Dilute Solution Routes to Various Controllable Morphologies of MCM-41 Silica with a Basic Medium. Chem. Mater..

[54] Nooney R.I., Thirunavukkarasu D., Yimei C., Josephs R., Ostafin A.E. (2002). Synthesis of Nanoscale Mesoporous Silica Spheres with Controlled Particle Size. Chem. Mater..

[55] Wu C.-G., Bein T. (1996). Microwave Synthesis of Molecular Sieve MCM-41. Chem. Commun..

[56] Zhao D., Feng J., Huo Q., Melosh N., Fredrickson G.H., Chmelka B.F., Stucky G.D. (1998). Triblock Copolymer Syntheses of Mesoporous Silica with Periodic 50 to 300 Angstrom Pores. Science.

[57] Allen L.H., Matijevíc E., Meites L. (1971). Exchange of Na+ for the Silanolic Protons of Silica. J. Inorg. Nucl. Chem..

[58] Lin H.P., Mou C.Y. (2002). Structural and Morphological Control of Cationic Surfactant-Templated Mesoporous Silica. Acc. Chem. Res..

[59] Vallet-Regi M., Rámila A., Del Real R.P., Pérez-Pariente J. (2001). A New Property of MCM-41: Drug Delivery System. Chem. Mater..

[60] Yang H., Vovk G., Coombs N., Sokolov I., Ozin G.A. (1998). Synthesis of Mesoporous Silica Spheres under Quiescent Aqueous Acidic Conditions. J. Mater. Chem..

[61] Voegtlin A.C., Matijasic A., Patarin J., Sauerland C., Grillet Y., Huve L. (1997). Room-Temperature Synthesis of Silicate Mesoporous MCM-41-Type Materials: Influence of the Synthesis PH on the Porosity of the Materials Obtained. Microporous Mater..

[62] Chiang Y.-D., Lian H.-Y., Leo S.-Y., Wang S.-G., Yamauchi Y., Wu K.C.-W. (2011). Controlling Particle Size and Structural Properties of Mesoporous Silica Nanoparticles Using the Taguchi Method. J. Phys. Chem. C.

[63] Mehmood A., Ghafar H., Yaqoob S., Gohar U.F., Ahmad B. (2017). Mesoporous Silica Nanoparticles: A Review. J. Dev. Drugs.

[64] Slowing I.I., Vivero-Escoto J.L., Trewyn B.G., Lin V.S.Y. (2010). Mesoporous Silica Nanoparticles: Structural Design and Applications. J. Mater. Chem..

[65] Kresge C.T., Leonowicz M.E., Roth W.J., Vartuli J.C., Beck J.S. (1992). Ordered Mesoporous Molecular Sieves Synthesized by a Liquid-Crystal Template Mechanism. Nature.

[66] Huo Q., Margolese D.I., Ciesla U., Feng P., Gier T.E., Sieger P., Leon R., Petroff P.M., Schüth F., Stucky G.D. (1994). Generalized Synthesis of Periodic Surfactant/Inorganic Composite Materials. Nature.

[67] Tanev P.T., Pinnavaia T.J. (1995). A Neutral Templating Route to Mesoporous Molecular Sieves. Science.

[68] Bagshaw S.A., Prouzet E., Pinnavaia T.J. (1995). Templating of Mesoporous Molecular Sieves by Nonionic Polyethylene Oxide Surfactants. Science.

[69] Lin H.Y., Chen Y.W. (2005). Preparation of Spherical Hexagonal Mesoporous Silica. J. Porous Mater..

[70] Mai W.X., Meng H. (2013). Mesoporous Silica Nanoparticles: A Multifunctional Nano Therapeutic System. Integr. Biol..

[71] Martin T., Galarneau A., Di Renzo F., Fajula F., Plee D. (2002). Morphological Control of MCM-41 by Pseudomorphic Synthesis. Angew. Chem. Int. Ed..

[72] Szegedi Á., Kónya Z., Méhn D., Solymár E., Pál-Borbély G., Horváth Z.E., Biró L.P., Kiricsi I. (2004). Spherical Mesoporous MCM-41 Materials Containing Transition Metals: Synthesis and Characterization. Appl. Catal. A Gen..

[73] Yano K., Fukushima Y. (2004). Synthesis of Mono-Dispersed Mesoporous Silica Spheres with Highly Ordered Hexagonal Regularity Using Conventional Alkyltrimethylammonium Halide as a Surfactant. J. Mater. Chem..

[74] Nandiyanto A.B.D., Kim S.-G., Iskandar F., Okuyama K. (2009). Synthesis of Spherical Mesoporous Silica Nanoparticles with Nanometer-Size Controllable Pores and Outer Diameters. Microporous Mesoporous Mater..

[75] Jafarzadeh M., Rahman I.A., Sipaut C.S. (2009). Synthesis of Silica Nanoparticles by Modified Sol-Gel Process: The Effect of Mixing Modes of the Reactants and Drying Techniques. J. Sol-Gel Sci. Technol..

[76] Lindberg R., Sjöblom J., Sundholm G. (1995). Preparation of Silica Particles Utilizing the Sol-Gel and the Emulsion-Gel Processes. Colloids Surf. A Physicochem. Eng. Asp..

[77] Van Blaaderen A., Van Geest J., Vrij A. (1992). Monodisperse Colloidal Silica Spheres from Tetraalkoxysilanes: Particle Formation and Growth Mechanism. J. Colloid Interface Sci..

[78] Gu J., Fan W., Shimojima A., Okubo T. (2007). Organic-Inorganic Mesoporous Nanocarriers Integrated with Biogenic Ligands. Small.

[79] Huh S., Wiench J.W., Yoo J.-C., Pruski M., Lin V.S.-Y. (2003). Organic Functionalization and Morphology Control of Mesoporous Silicas via a Co-Condensation Synthesis Method. Chem. Mater..

[80] Caruso F., Caruso R.A., Möhwald H. (1999). Production of Hollow Microspheres from Nanostructured Composite Particles. Chem. Mater..

[81] Yamada H., Urata C., Aoyama Y., Osada S., Yamauchi Y., Kuroda K. (2012). Preparation of Colloidal Mesoporous Silica Nanoparticles with Different Diameters and Their Unique Degradation Behavior in Static Aqueous Systems. Chem. Mater..

[82] He Q., Cui X., Cui F., Guo L., Shi J. (2009). Size-Controlled Synthesis of Monodispersed Mesoporous Silica Nano-Spheres under a Neutral Condition. Microporous Mesoporous Mater..

[83] Wu S.H., Hung Y., Mou C.Y. (2011). Mesoporous Silica Nanoparticles as Nanocarriers. Chem. Commun..

[84] Itoh Y., Matsusaki M., Kida T., Akashi M. (2004). Preparation of Biodegradable Hollow Nanocapsules by Silica Template Method. Chem. Lett..

[85] Zhang H., Zhou Y., Li Y., Bandosz T.J., Akins D.L. (2012). Synthesis of Hollow Ellipsoidal Silica Nanostructures Using a Wet-Chemical Etching Approach. J. Colloid Interface Sci..

[86] Tanev P.T., Pinnavaia T.J. (1996). Biomimetic Templating of Porous Lamellar Silicas by Vesicular Surfactant Assemblies. Science.

[87] Li W., Coppens M. (2005). Synthesis and Characterization of Stable Hollow Ti-Silica. Chem. Mater..

[88] Lin Y.S., Wu S.H., Tseng C.T., Hung Y., Chang C., Mou C.Y. (2009). Synthesis of Hollow Silica Nanospheres with a Microemulsion as the Template. Chem. Commun..

[89] Croissant J.G., Fatieiev Y., Almalik A., Khashab N.M. (2018). Mesoporous Silica and Organosilica Nanoparticles: Physical Chemistry, Biosafety, Delivery Strategies, and Biomedical Applications. Adv. Healthc. Mater..

[90] Poscher V., Salinas Y. (2020). Trends in Degradable Mesoporous Organosilica-Based Nanomaterials for Controlling Drug Delivery: A Mini Review. Materials.

[91] Díaz Morales U.M., Corma Canós A. (2018). Organic-Inorganic Hybrid Materials: MultiFunctional Solids for Multi-Step Reaction Processes. Chem.—A Eur. J..

[92] Erigoni A., Diaz U. (2021). Porous Silica-Based Organic-Inorganic Hybrid Catalysts: A Review. Catalysts.

[93] Croissant J.G., Cattoën X., Wong Chi Man M., Durand J.-O., Khashab N.M. (2015). Syntheses and Applications of Periodic Mesoporous Organosilica Nanoparticles. Nanoscale.

[94] Chinnathambi S., Tamanoi F. (2020). Recent Development to Explore the Use of Biodegradable Periodic Mesoporous Organosilica (BPMO) Nanomaterials for Cancer Therapy. Pharmaceutics.

[95] Yu L., Chen Y., Lin H., Du W., Chen H., Shi J. (2018). Ultrasmall Mesoporous Organosilica Nanoparticles: Morphology Modulations and Redox-Responsive Biodegradability for Tumor-Specific Drug Delivery. Biomaterials.

[96] Sacks M.D., Tseng T.-Y. (1984). Preparation of SiO2 Glass from Model Powder Compacts: I, Formation and Characterization of Powders, Suspensions, and Green Compacts. J. Am. Ceram. Soc..

[97] Unger K.K., Kumar D., Grün M., Büchel G., Lüdtke S., Adam T., Schumacher K., Renker S. (2000). Synthesis of Spherical Porous Silicas in the Micron and Submicron Size Range: Challenges and Opportunities for Miniaturized High-Resolution Chromatographic and Electrokinetic Separations. J. Chromatogr. A.

[98] Míguez H., López C., Meseguer F., Blanco A., Vázquez L., Mayoral R., Ocaña M., Fornés V., Mifsud A. (1997). Photonic Crystal Properties of Packed Submicrometric SiO2 Spheres. Appl. Phys. Lett..

[99] Kochergin Y.S., Villa K., Nemeškalová A., Kuchař M., Pumera M. (2021). Hybrid Inorganic-Organic Visible-Light-Driven Microrobots Based on Donor-Acceptor Organic Polymer for Degradation of Toxic Psychoactive Substances. ACS Nano.

[100] Hortelao A.C., Simó C., Guix M., Guallar-Garrido S., Julián E., Vilela D., Rejc L., Ramos-Cabrer P., Cossío U., Gómez-Vallejo V. (2021). Swarming Behavior and in Vivo Monitoring of Enzymatic Nanomotors within the Bladder. Sci. Robot..

[101] Lu J., Liong M., Sherman S., Xia T., Kovochich M., Nel A.E., Zink J.I., Tamanoi F. (2007). Mesoporous Silica Nanoparticles for Cancer Therapy: Energy-Dependent Cellular Uptake and Delivery of Paclitaxel to Cancer Cells. Nanobiotechnology.

[102] Sung H., Ferlay J., Siegel R.L., Laversanne M., Soerjomataram I., Jemal A., Bray F. (2021). Global Cancer Statistics 2020: GLOBOCAN Estimates of Incidence and Mortality Worldwide for 36 Cancers in 185 Countries. CA Cancer J. Clin..

[103] Garcia-Oliveira P., Otero P., Pereira A.G., Chamorro F., Carpena M., Echave J., Fraga-Corral M., Simal-Gandara J., Prieto M.A. (2021). Status and Challenges of Plant-Anticancer Compounds in Cancer Treatment. Pharmaceuticals.

[104] Papafilippou L., Claxton A., Dark P., Kostarelos K., Hadjidemetriou M. (2021). Nanotools for Sepsis Diagnosis and Treatment. Adv. Healthc. Mater..

[105] Wang J.T.-W., Klippstein R., Martincic M., Pach E., Feldman R., Šefl M., Michel Y., Asker D., Sosabowski J.K., Kalbac M. (2020). Neutron Activated ^153^Sm Sealed in Carbon Nanocapsules for in Vivo Imaging and Tumor Radiotherapy. ACS Nano.

[106] Wang Y., Zhao Q., Han N., Bai L., Li J., Liu J., Che E., Hu L., Zhang Q., Jiang T. (2015). Mesoporous Silica Nanoparticles in Drug Delivery and Biomedical Applications. Nanomed. Nanotechnol. Biol. Med..

[107] Martincic M., Tobias G. (2015). Filled Carbon Nanotubes in Biomedical Imaging and Drug Delivery. Expert Opin. Drug Deliv..

[108] Baeza A., Manzano M., Colilla M., Vallet-Regí M. (2016). Recent Advances in Mesoporous Silica Nanoparticles for Antitumor Therapy: Our Contribution. Biomater. Sci..

[109] Koohi Moftakhari Esfahani M., Alavi S.E., Cabot P.J., Islam N., Izake E.L. (2022). Application of Mesoporous Silica Nanoparticles in Cancer Therapy and Delivery of Repurposed Anthelmintics for Cancer Therapy. Pharmaceutics.

[110] Bukara K., Schueller L., Rosier J., Martens M.A., Daems T., Verheyden L., Eelen S., Van Speybroeck M., Libanati C., Martens J.A. (2016). Ordered Mesoporous Silica to Enhance the Bioavailability of Poorly Water-Soluble Drugs: Proof of Concept in Man. Eur. J. Pharm. Biopharm..

[111] Rastinehad A.R., Anastos H., Wajswol E., Winoker J.S., Sfakianos J.P., Doppalapudi S.K., Carrick M.R., Knauer C.J., Taouli B., Lewis S.C. (2019). Gold Nanoshell-Localized Photothermal Ablation of Prostate Tumors in a Clinical Pilot Device Study. Proc. Natl. Acad. Sci. USA.

[112] Zanoni D.K., Stambuk H.E., Madajewski B., Montero P.H., Matsuura D., Busam K.J., Ma K., Turker M.Z., Sequeira S., Gonen M. (2021). Use of Ultrasmall Core-Shell Fluorescent Silica Nanoparticles for Image-Guided Sentinel Lymph Node Biopsy in Head and Neck Melanoma: A Nonrandomized Clinical Trial. JAMA Netw. Open.

[113] Bostonscientific. https://www.bostonscientific.com/en-US/medical-specialties/interventional-radiology/interventional-oncology/cancer-therapies-ablation/therasphere.html.

[114] Jafari S., Derakhshankhah H., Alaei L., Fattahi A., Varnamkhasti B.S., Saboury A.A. (2019). Mesoporous Silica Nanoparticles for Therapeutic/Diagnostic Applications. Biomed. Pharmacother..

[115] Vazquez N.I., Gonzalez Z., Ferrari B., Castro Y. (2017). Synthesis of Mesoporous Silica Nanoparticles by Sol-Gel as Nanocontainer for Future Drug Delivery Applications. Bol. la Soc. Esp. Ceram. y Vidr..

[116] Zhang Y., Lou J., Williams G.R., Ye Y., Ren D., Shi A., Wu J., Chen W., Zhu L.-M. (2022). Cu^2+^-Chelating Mesoporous Silica Nanoparticles for Synergistic Chemotherapy/Chemodynamic Therapy. Pharmaceutics.

[117] Tarannum M., Holtzman K., Dréau D., Mukherjee P., Vivero-Escoto J.L. (2022). Nanoparticle Combination for Precise Stroma Modulation and Improved Delivery for Pancreatic Cancer. J. Control. Release.

[118] Shahidi M., Abazari O., Dayati P., Bakhshi A., Zavarreza J., Modarresi M.H., Haghiralsadat F., Rahmanian M., Naghib S.M., Tofighi D. (2022). Multicomponent SiRNA/MiRNA-Loaded Modified Mesoporous Silica Nanoparticles Targeted Bladder Cancer for a Highly Effective Combination Therapy. Front. Bioeng. Biotechnol..

[119] Gu Y., Fei Z. (2022). Mesoporous Silica Nanoparticles Loaded with Resveratrol Are Used for Targeted Breast Cancer Therapy. J. Oncol..

[120] Song Q., Yang W., Deng X., Zhang Y., Li J., Xing X., Chen W., Liu W., Hu H., Zhang Y. (2022). Platinum-Based Nanocomposites Loaded with MTH1 Inhibitor Amplify Oxidative Damage for Cancer Therapy. Colloids Surf. B Biointerfaces.

[121] Li H., Zhu J., Xu Y.-W., Mou F.-F., Shan X.-L., Wang Q.-L., Liu B.-N., Ning K., Liu J.-J., Wang Y.-C. (2022). Notoginsenoside R1-Loaded Mesoporous Silica Nanoparticles Targeting the Site of Injury through Inflammatory Cells Improves Heart Repair after Myocardial Infarction. Redox Biol..

[122] Mao S., Wang S., Niu Y., Wu J., Jia P., Zheng J., Dong Y. (2022). Induction of Cartilage Regeneration by Nanoparticles Loaded with Dentin Matrix Extracted Proteins. Tissue Eng. Part A.

[123] Wang H., Chang X., Ma Q., Sun B., Li H., Zhou J., Hu Y., Yang X., Li J., Chen X. (2023). Bioinspired Drug-Delivery System Emulating the Natural Bone Healing Cascade for Diabetic Periodontal Bone Regeneration. Bioact. Mater..

[124] Chen X., Zhu X., Hu Y., Yuan W., Qiu X., Jiang T., Xia C., Xiong L., Li F., Gao Y. (2020). EDTA-Modified 17β-Estradiol-Laden Upconversion Nanocomposite for Bone-Targeted Hormone Replacement Therapy for Osteoporosis. Theranostics.

[125] Díaz-García D., Ferrer-Donato Á., Méndez-Arriaga J.M., Cabrera-Pinto M., Díaz-Sánchez M., Prashar S., Fernandez-Martos C.M., Gómez-Ruiz S. (2022). Design of Mesoporous Silica Nanoparticles for the Treatment of Amyotrophic Lateral Sclerosis (ALS) with a Therapeutic Cocktail Based on Leptin and Pioglitazone. ACS Biomater. Sci. Eng..

[126] Ribeiro T.D.C., Sábio R.M., Luiz M.T., de Souza L.C., Fonseca-Santos B., Cides da Silva L.C., Fantini M.C.D.A., Planeta C.D.S., Chorilli M. (2022). Curcumin-Loaded Mesoporous Silica Nanoparticles Dispersed in Thermo-Responsive Hydrogel as Potential Alzheimer Disease Therapy. Pharmaceutics.

[127] Chen G., Bai Y., Li Z., Wang F., Fan X., Zhou X. (2020). Bacterial Extracellular Vesicle-Coated Multi-Antigenic Nanovaccines Protect against Drug-Resistant Staphylococcus Aureus Infection by Modulating Antigen Processing and Presentation Pathways. Theranostics.

[128] Ahmed N., Fessi H., Elaissari A. (2012). Theranostic Applications of Nanoparticles in Cancer. Drug Discov. Today.

[129] Blanco E., Shen H., Ferrari M. (2015). Principles of Nanoparticle Design for Overcoming Biological Barriers to Drug Delivery. Nat. Biotechnol..

[130] Liu W., Zhang L., Dong Z., Liu K., He H., Lu Y., Wu W., Qi J. (2022). Rod-like Mesoporous Silica Nanoparticles Facilitate Oral Drug Delivery via Enhanced Permeation and Retention Effect in Mucus. Nano Res..

[131] Xi Z., Zhang W., Fei Y., Cui M., Xie L., Chen L., Xu L. (2020). Evaluation of the Solid Dispersion System Engineered from Mesoporous Silica and Polymers for the Poorly Water Soluble Drug Indomethacin: In Vitro and in Vivo. Pharmaceutics.

[132] Fahmy H.M., Fathy M.M., Abd-elbadia R.A., Elshemey W.M. (2019). Targeting of Thymoquinone-Loaded Mesoporous Silica Nanoparticles to Different Brain Areas: In Vivo Study. Life Sci..

[133] Niu X., Wang X., Niu B., Li G., Yang X., Wang Y., Li G. (2021). Novel Imb16-4 Compound Loaded into Silica Nanoparticles Exhibits Enhanced Oral Bioavailability and Increased Anti-Liver Fibrosis in Vitro. Molecules.

[134] Zhang Q., Pei Q., Yang J., Guo S., Yang A., Qian Y., Li C., Feng Q., Lv H., Zhou X. (2022). Vascularized Nanocomposite Hydrogel Mechanically Reinforced by Polyelectrolyte-Modified Nanoparticles. J. Mater. Chem. B.

[135] Sun J.G., Jiang Q., Zhang X.P., Shan K., Liu B.H., Zhao C., Yan B. (2019). Mesoporous Silica Nanoparticles as a Delivery System for Improving Antiangiogenic Therapy. Int. J. Nanomed..

[136] Mehmood Y., Khan I.U., Shahzad Y., Khan R.U., Iqbal M.S., Khan H.A., Khalid I., Yousaf A.M., Khalid S.H., Asghar S. (2020). In-Vitro and in-Vivo Evaluation of Velpatasvir-Loaded Mesoporous Silica Scaffolds. A Prospective Carrier for Drug Bioavailability Enhancement. Pharmaceutics.

[137] Chen Z., Han L., Liu C., Du Y., Hu X., Du G., Shan C., Yang K., Wang C., Li M. (2018). A Rapid Hemostatic Sponge Based on Large, Mesoporous Silica Nanoparticles and: N -Alkylated Chitosan. Nanoscale.

[138] Liu Q., Zhang Y., Huang J., Xu Z., Li X., Yang J., Huang H., Tang S., Chai Y., Lin J. (2022). Mesoporous Silica-Coated Silver Nanoparticles as Ciprofloxacin/SiRNA Carriers for Accelerated Infected Wound Healing. J. Nanobiotechnol..

[139] Dong W., Wen J., Li Y., Wang C., Sun S., Shang D. (2020). Targeted Antimicrobial Peptide Delivery in Vivo to Tumor with near Infrared Photoactivated Mesoporous Silica Nanoparticles. Int. J. Pharm..

[140] Xu J., Zhang J., Song J., Liu Y., Li J., Wang X., Tang R. (2022). Construction of Multifunctional Mesoporous Silicon Nano-Drug Delivery System and Study of Dual Sensitization of Chemo-Photodynamic Therapy in Vitro and in Vivo. J. Colloid Interface Sci..

[141] Lin M., Yao W., Xiao Y., Dong Z., Huang W., Zhang F., Zhou X., Liang M. (2021). Resveratrol-Modified Mesoporous Silica Nanoparticle for Tumor-Targeted Therapy of Gastric Cancer. Bioengineered.

[142] Kundu M., Sadhukhan P., Ghosh N., Ghosh S., Chatterjee S., Das J., Brahmachari G., Sil P.C. (2021). In Vivo Therapeutic Evaluation of a Novel Bis-Lawsone Derivative against Tumor Following Delivery Using Mesoporous Silica Nanoparticle Based Redox-Responsive Drug Delivery System. Mater. Sci. Eng. C.

[143] Liao T., Liu C., Ren J., Chen H., Kuang Y., Jiang B., Chen J., Sun Z., Li C. (2021). A Chitosan/Mesoporous Silica Nanoparticle-Based Anticancer Drug Delivery System with a “Tumor-Triggered Targeting” Property. Int. J. Biol. Macromol..

[144] Li Y.S., Wu H.H., Jiang X.C., Zhang T.Y., Zhou Y., Huang L.L., Zhi P., Tabata Y., Gao J.Q. (2021). Active Stealth and Self-Positioning Biomimetic Vehicles Achieved Effective Antitumor Therapy. J. Control. Release.

[145] Zhang D., Zhang W., Wu X., Li Q., Mu Z., Sun F., Zhang M., Liu G., Hu L. (2021). Dual Modal Imaging-Guided Drug Delivery System for Combined Chemo-Photothermal Melanoma Therapy. Int. J. Nanomed..

[146] Ding Z., Wang D., Shi W., Yang X., Duan S., Mo F., Hou X., Liu A., Lu X. (2020). In Vivo Targeting of Liver Cancer with Tissue-and Nuclei-Specific Mesoporous Silica Nanoparticle-Based Nanocarriers in Mice. Int. J. Nanomed..

[147] Babaei M., Abnous K., Taghdisi S.M., Taghavi S., Saljooghi A.S., Ramezani M., Alibolandi M. (2020). Targeted Rod-Shaped Mesoporous Silica Nanoparticles for the Co-Delivery of Camptothecin and Survivin ShRNA in to Colon Adenocarcinoma In Vitro and In Vivo. Eur. J. Pharm. Biopharm..

[148] Kundu M., Chatterjee S., Ghosh N., Manna P., Das J., Sil P.C. (2020). Tumor Targeted Delivery of Umbelliferone via a Smart Mesoporous Silica Nanoparticles Controlled-Release Drug Delivery System for Increased Anticancer Efficiency. Mater. Sci. Eng. C.

[149] Choi J.Y., Gupta B., Ramasamy T., Jeong J.-H., Jin S.G., Choi H.-G., Yong C.S., Kim J.O. (2018). PEGylated Polyaminoacid-Capped Mesoporous Silica Nanoparticles for Mitochondria-Targeted Delivery of Celastrol in Solid Tumors. Colloids Surf. B Biointerfaces.

[150] Li Z., Guo J., Qi G., Zhang M., Hao L. (2022). PH-Responsive Drug Delivery and Imaging Study of Hybrid Mesoporous Silica Nanoparticles. Molecules.

[151] Sivasubramanian M., Chu C.-H., Cheng S.-H., Chen N.-T., Chen C.-T., Chuang Y.C., Yu H., Chen Y.-L., Liao L.-D., Lo L.-W. (2022). Multimodal Magnetic Resonance and Photoacoustic Imaging of Tumor-Specific Enzyme-Responsive Hybrid Nanoparticles for Oxygen Modulation. Front. Bioeng. Biotechnol..

[152] Zhang W., Lv Z., Zhang Y., Gopinath S.C.B., Yuan Y., Huang D., Miao L. (2022). Targeted Diagnosis, Therapeutic Monitoring, and Assessment of Atherosclerosis Based on Mesoporous Silica Nanoparticles Coated with CRGD-Platelets. Oxid. Med. Cell. Longev..

[153] Dou Y., Zhang Y., Lin C., Han R., Wang Y., Wu D., Zheng J., Lu C., Tang L., He Y. (2022). PH-Responsive Theranostic Nanoplatform of Ferrite and Ceria Co-Engineered Nanoparticles for Anti-Inflammatory. Front. Bioeng. Biotechnol..

[154] El-Shahawy A.A.G., Zohery M., El-Dek S.I. (2022). Theranostics Platform of Abemaciclib Using Magnetite@silica@chitosan Nanocomposite. Int. J. Biol. Macromol..

[155] Lu J., Ni C., Huang J., Liu Y., Tao Y., Hu P., Wang Y., Zheng S., Shi M. (2021). Biocompatible Mesoporous Silica-Polydopamine Nanocomplexes as MR/Fluorescence Imaging Agent for Light-Activated Photothermal-Photodynamic Cancer Therapy In Vivo. Front. Bioeng. Biotechnol..

[156] Zhang Y., Qian C., Li D., Zhao L. (2022). Rational Surface Modification of Gadolinium Borate Nanoparticles Enhancing Colloidal Stability in Physiological Media for Potential Neutron Capture Therapy and Magnetic Resonance Imaging. Colloids Surf. B Biointerfaces.

[157] Li Z., Guo J., Zhang M., Li G., Hao L. (2022). Gadolinium-Coated Mesoporous Silica Nanoparticle for Magnetic Resonance Imaging. Front. Chem..

[158] Wang J., Zha M., Zhao H., Yue W., Wu D., Li K. (2022). Detection of Kidney Dysfunction through In Vivo Magnetic Resonance Imaging with Renal-Clearable Gadolinium Nanoprobes. Anal. Chem..

[159] Wu M., Li X., Guo Q., Li J., Xu G., Li G., Wang J., Zhang X. (2021). Magnetic Mesoporous Silica Nanoparticles-Aided Dual MR/NIRF Imaging to Identify Macrophage Enrichment in Atherosclerotic Plaques. Nanomed. Nanotechnol. Biol. Med..

[160] Du D., Fu H.-J., Ren W., Li X.-L., Guo L.-H. (2020). PSA Targeted Dual-Modality Manganese Oxide–Mesoporous Silica Nanoparticles for Prostate Cancer Imaging. Biomed. Pharmacother..

[161] Li J., Zhu F., Lou K., Tian H., Luo Q., Dang Y., Liu X., Wang P., Wu L. (2022). Tumor Microenvironment Enhanced NIR II Fluorescence Imaging for Tumor Precise Surgery Navigation via Tetrasulfide Mesoporous Silica-Coated Nd-Based Rare-Earth Nanocrystals. Mater. Today Bio.

[162] Li Y., Zhu B., Han W., Tang W., Duan X. (2022). A Bright Chemiluminescence Conjugated Polymer-Mesoporous Silica Nanoprobe for Imaging of Colonic Tumors in Vivo. Analyst.

[163] Shi S., Chen F., Goel S., Graves S.A., Luo H., Theuer C.P., Engle J.W., Cai W. (2018). In Vivo Tumor-Targeted Dual-Modality PET/Optical Imaging with a Yolk/Shell-Structured Silica Nanosystem. Nano-Micro Lett..

[164] Jeong H.J., Yoo R.J., Kim J.K., Kim M.H., Park S.H., Kim H., Lim J.W., Do S.H., Lee K.C., Lee Y.J. (2019). Macrophage Cell Tracking PET Imaging Using Mesoporous Silica Nanoparticles via in Vivo Bioorthogonal F-18 Labeling. Biomaterials.

[165] Ellison P.A., Chen F., Goel S., Barnhart T.E., Nickles R.J., DeJesus O.T., Cai W. (2017). Intrinsic and Stable Conjugation of Thiolated Mesoporous Silica Nanoparticles with Radioarsenic. ACS Appl. Mater. Interfaces.

[166] Zou R., Gao Y., Zhang Y., Jiao J., Wong K.-L., Wang J. (2021). (68)Ga-Labeled Magnetic-NIR Persistent Luminescent Hybrid Mesoporous Nanoparticles for Multimodal Imaging-Guided Chemotherapy and Photodynamic Therapy. ACS Appl. Mater. Interfaces.

[167] Xu C., Chen F., Valdovinos H.F., Jiang D., Goel S., Yu B., Sun H., Barnhart T.E., Moon J.J., Cai W. (2018). Bacteria-like Mesoporous Silica-Coated Gold Nanorods for Positron Emission Tomography and Photoacoustic Imaging-Guided Chemo-Photothermal Combined Therapy. Biomaterials.

[168] Dogra P., Adolphi N.L., Wang Z., Lin Y.-S., Butler K.S., Durfee P.N., Croissant J.G., Noureddine A., Coker E.N., Bearer E.L. (2018). Establishing the Effects of Mesoporous Silica Nanoparticle Properties on in Vivo Disposition Using Imaging-Based Pharmacokinetics. Nat. Commun..

[169] Argyo C., Weiss V., Bräuchle C., Bein T. (2014). Multifunctional Mesoporous Silica Nanoparticles as a Universal Platform for Drug Delivery. Chem. Mater..

[170] Zhou Y., Yang S., Guo J., Dong H., Yin K., Huang W.T., Yang R. (2020). In Vivo Imaging of Hypoxia Associated with Inflammatory Bowel Disease by a Cytoplasmic Protein-Powered Fluorescence Cascade Amplifier. Anal. Chem..

[171] Taylor K.M.L., Kim J.S., Rieter W.J., An H., Lin W., Lin W. (2008). Mesoporous Silica Nanospheres as Highly Efficient MRI Contrast Agents. J. Am. Chem. Soc..

[172] Grzelak J., Gázquez J., Grayston A., Teles M., Herranz F., Roher N., Rosell A., Roig A., Gich M. (2022). Magnetic Mesoporous Silica Nanorods Loaded with Ceria and Functionalized with Fluorophores for Multimodal Imaging. ACS Appl. Nano Mater..

[173] Chen F., Goel S., Valdovinos H.F., Luo H., Hernandez R., Barnhart T.E., Cai W. (2015). In Vivo Integrity and Biological Fate of Chelator-Free Zirconium-89-Labeled Mesoporous Silica Nanoparticles. ACS Nano.

[174] Mody K.T., Popat A., Mahony D., Cavallaro A.S., Yu C., Mitter N. (2013). Mesoporous Silica Nanoparticles as Antigen Carriers and Adjuvants for Vaccine Delivery. Nanoscale.

[175] de Pádua Oliveira D.C., de Barros A.L.B., Belardi R.M., de Goes A.M., de Oliveira Souza B.K., Soares D.C.F. (2016). Mesoporous Silica Nanoparticles as a Potential Vaccine Adjuvant against Schistosoma Mansoni. J. Drug Deliv. Sci. Technol..

[176] Zhao L., Seth A., Wibowo N., Zhao C.-X., Mitter N., Yu C., Middelberg A.P.J. (2014). Nanoparticle Vaccines. Vaccine.

[177] Hong X., Zhong X., Du G., Hou Y., Zhang Y., Zhang Z., Gong T., Zhang L., Sun X. (2022). The Pore Size of Mesoporous Silica Nanoparticles Regulates Their Antigen Delivery Efficiency. Sci. Adv..

[178] Niculescu V.-C. (2020). Mesoporous Silica Nanoparticles for Bio-Applications. Front. Mater..

[179] Mody K.T., Mahony D., Zhang J., Cavallaro A.S., Zhang B., Popat A., Mahony T.J., Yu C., Mitter N. (2014). Silica Vesicles as Nanocarriers and Adjuvants for Generating Both Antibody and T-Cell Mediated Immune Resposes to Bovine Viral Diarrhoea Virus E2 Protein. Biomaterials.

[180] Montalvo-Quirós S., Vallet-Regí M., Palacios A., Anguita J., Prados-Rosales R.C., González B., Luque-Garcia J.L. (2020). Mesoporous Silica Nanoparticles as a Potential Platform for Vaccine Development against Tuberculosis. Pharmaceutics.

[181] Qiao L., Chen M., Li S., Hu J., Gong C., Zhang Z., Cao X. (2021). A Peptide-Based Subunit Candidate Vaccine against SARS-CoV-2 Delivered by Biodegradable Mesoporous Silica Nanoparticles Induced High Humoral and Cellular Immunity in Mice. Biomater. Sci..

[182] Liang H., Jin C., Ma L., Feng X., Deng X., Wu S., Liu X., Yang C. (2019). Accelerated Bone Regeneration by Gold-Nanoparticle-Loaded Mesoporous Silica through Stimulating Immunomodulation. ACS Appl. Mater. Interfaces.

[183] Jia Y., Zhang P., Sun Y., Kang Q., Xu J., Zhang C., Chai Y. (2019). Regeneration of Large Bone Defects Using Mesoporous Silica Coated Magnetic Nanoparticles during Distraction Osteogenesis. Nanomedicine.

[184] Hench L.L. (2006). The Story of Bioglass. J. Mater. Sci. Mater. Med..

[185] Deilmann L., Winter O., Cerrutti B., Bradtmüller H., Herzig C., Limbeck A., Lahayne O., Hellmich C., Eckert H., Eder D. (2020). Effect of Boron Incorporation on the Bioactivity, Structure, and Mechanical Properties of Ordered Mesoporous Bioactive Glasses. J. Mater. Chem. B.

[186] Vallet-Regi M., Salinas A.J. (2021). Mesoporous Bioactive Glasses for Regenerative Medicine. Mater. Today Bio.

[187] Tavares M.T., Santos S.C., Custódio C.A., Farinha J.P.S., Baleizão C., Mano J.F. (2021). Platelet Lysates-Based Hydrogels Incorporating Bioactive Mesoporous Silica Nanoparticles for Stem Cell Osteogenic Differentiation. Mater. Today Bio.

[188] Rosenholm J.M., Zhang J.X., Linden M., Sahlgren C. (2016). Mesoporous Silica Nanoparticles in Tissue Engineering—A Perspective. Nanomedicine.

[189] Mosquera J., García I., Henriksen-Lacey M., González G., Liz-Marzán L. (2018). Reducing Protein Corona Formation and Enhancing Colloidal Stability of Gold Nanoparticles by Capping with Silica Monolayers. Chem. Mater..

[190] Jiang X., Liu X., Yu Q., Shen W., Mei X., Tian H., Wu C. (2022). Functional Resveratrol-Biodegradable Manganese Doped Silica Nanoparticles for the Spinal Cord Injury Treatment. Mater. Today Bio.

[191] Li T., Shi S., Goel S., Shen X., Xie X., Chen Z., Zhang H., Li S., Qin X., Yang H. (2019). Recent Advancements in Mesoporous Silica Nanoparticles towards Therapeutic Applications for Cancer. Acta Biomater..

[192] Ahmadi E., Dehghannejad N., Hashemikia S., Ghasemnejad M., Tabebordbar H. (2014). Synthesis and Surface Modification of Mesoporous Silica Nanoparticles and Its Application as Carriers for Sustained Drug Delivery. Drug Deliv..

[193] Tarn D., Ashley C.E., Xue M., Carnes E., Zink J.I., Brinker J. (2013). Mesoporous Silica Nanoparticle Nanocarriers. Acc. Chem. Res..

[194] Giret S., Man M.W.C., Carcel C. (2015). Mesoporous-Silica-Functionalized Nanoparticles for Drug Delivery. Chem.-A Eur. J..

[195] Baeza A., Colilla M., Vallet-Regí M. (2015). Advances in Mesoporous Silica Nanoparticles for Targeted Stimuli-Responsive Drug Delivery. Expert Opin. Drug Deliv..

[196] Kumar Khanna V. (2012). Targeted Delivery of Nanomedicines. ISRN Pharmacol..

[197] Devarajan P.V., Dawre S.M., Dutta R., Devarajan P.V., Jain S. (2015). Infectious Diseases: Need for Targeted Drug Delivery BT-Targeted Drug Delivery: Concepts and Design.

[198] Nel A.E., Mädler L., Velegol D., Xia T., Hoek E.M.V., Somasundaran P., Klaessig F., Castranova V., Thompson M. (2009). Understanding Biophysicochemical Interactions at the Nano-Bio Interface. Nat. Mater..

[199] Li Y., Zhang Y., Wang L., Wang P., Xue Y., Li X., Qiao X., Zhang X., Xu T., Liu G. (2017). Autophagy Impairment Mediated by S-Nitrosation of ATG4B Leads to Neurotoxicity in Response to Hyperglycemia. Autophagy.

[200] He X., Nie H., Wang K., Tan W., Wu X., Zhang P. (2008). In Vivo Study of Biodistribution and Urinary Excretion of Surface-Modified Silica Nanoparticles. Anal. Chem..

[201] Cui Y., Dong H., Cai X., Wang D., Li Y. (2012). Mesoporous Silica Nanoparticles Capped with Disulfide-Linked PEG Gatekeepers for Glutathione-Mediated Controlled Release. ACS Appl. Mater. Interfaces.

[202] Tam V.H., Sosa C., Liu R., Yao N., Priestley R.D. (2016). Nanomedicine as a Non-Invasive Strategy for Drug Delivery across the Blood Brain Barrier. Int. J. Pharm..

[203] Kwon S., Singh R.K., Perez R.A., Neel E.A.A., Kim H.W., Chrzanowski W. (2013). Silica-Based Mesoporous Nanoparticles for Controlled Drug Delivery. J. Tissue Eng..

[204] Vartuli J.C., Shih S.S., Kresge C.T., Beck J.S., Bonneviot L., Béland F., Danumah C., Giasson S., Kaliaguine S. (1998). Potential Applications for M41S Type Mesoporous Molecular Sieves. Mesoporous Molecular Sieves 1998.

[205] Cauda V., Schlossbauer A., Bein T. (2010). Bio-Degradation Study of Colloidal Mesoporous Silica Nanoparticles: Effect of Surface Functionalization with Organo-Silanes and Poly(Ethylene Glycol). Microporous Mesoporous Mater..

[206] Oh C., Do Ki C., Young Chang J., Oh S.G. (2005). Preparation of PEG-Grafted Silica Particles Using Emulsion Method. Mater. Lett..

[207] Zalipsky S., Harris J.M. (1997). Introduction to Chemistry and Biological Applications of Poly(Ethylene Glycol). ACS Symp. Ser..

[208] Naahidi S., Jafari M., Edalat F., Raymond K., Khademhosseini A., Chen P. (2013). Biocompatibility of Engineered Nanoparticles for Drug Delivery. J. Control. Release.

[209] Jokerst J.V., Lobovkina T., Zare R.N., Gambhir S.S. (2011). Nanoparticle PEGylation for Imaging and Therapy. Nanomedicine.

[210] Cauda V., Argyo C., Bein T. (2010). Impact of Different PEGylation Patterns on the Long-Term Bio-Stability of Colloidal Mesoporous Silica Nanoparticles. J. Mater. Chem..

[211] He Q., Zhang J., Shi J., Zhu Z., Zhang L., Bu W., Guo L., Chen Y. (2010). The Effect of PEGylation of Mesoporous Silica Nanoparticles on Nonspecific Binding of Serum Proteins and Cellular Responses. Biomaterials.

[212] Shen L., Pan S., Niu D., He J., Jia X., Hao J., Gu J., Zhao W., Li P., Li Y. (2019). Facile Synthesis of Organosilica-Capped Mesoporous Silica Nanocarriers with Selective Redox-Triggered Drug Release Properties for Safe Tumor Chemotherapy. Biomater. Sci..

[213] Feng L., Wang Y., Wang N., Ma Y. (2009). Preparation of Poly(Ethylene Glycol)-Grafted Silica Nanoparticles Using a Facile Esterification Condensation Method. Polym. Bull..

[214] Feng B., Hong R.Y., Wang L.S., Guo L., Li H.Z., Ding J., Zheng Y., Wei D.G. (2008). Synthesis of Fe_3_O_4_/APTES/PEG Diacid Functionalized Magnetic Nanoparticles for MR Imaging. Colloids Surf. A Physicochem. Eng. Asp..

[215] Zhu Y., Fang Y., Borchardt L., Kaskel S. (2011). PEGylated Hollow Mesoporous Silica Nanoparticles as Potential Drug Delivery Vehicles. Microporous Mesoporous Mater..

[216] Huang X., Li L., Liu T., Hao N., Liu H., Chen D., Tang F. (2011). The Shape Effect of Mesoporous Silica Nanoparticles on Biodistribution, Clearance, and Biocompatibility in Vivo. ACS Nano.

[217] Mohamed Isa E.D., Ahmad H., Abdul Rahman M.B., Gill M.R. (2021). Progress in Mesoporous Silica Nanoparticles as Drug Delivery Agents for Cancer Treatment. Pharmaceutics.

[218] Huang P., Lian D., Ma H., Gao N., Zhao L., Luan P., Zeng X. (2021). New Advances in Gated Materials of Mesoporous Silica for Drug Controlled Release. Chinese Chem. Lett..

[219] Gisbert-Garzarán M., Vallet-Regí M. (2021). Redox-Responsive Mesoporous Silica Nanoparticles for Cancer Treatment: Recent Updates. Nanomaterials.

[220] Alyassin Y., Sayed E.G., Mehta P., Ruparelia K., Arshad M.S., Rasekh M., Shepherd J., Kucuk I., Wilson P.B., Singh N. (2020). Application of Mesoporous Silica Nanoparticles as Drug Delivery Carriers for Chemotherapeutic Agents. Drug Discov. Today.

[221] Slowing I., Trewyn B.G., Lin V.S.Y. (2006). Effect of Surface Functionalization of MCM-41-Type Mesoporous Silica Nanoparticles on the Endocytosis by Human Cancer Cells. J. Am. Chem. Soc..

[222] Díez P., Lucena-Sánchez E., Escudero A., Llopis-Lorente A., Villalonga R., Martínez-Máñez R. (2021). Ultrafast Directional Janus Pt–Mesoporous Silica Nanomotors for Smart Drug Delivery. ACS Nano.

[223] Saadat M., Zahednezhad F., Zakeri-Milani P., Reza Heidari H., Shahbazi-Mojarrad J., Valizadeh H. (2019). Drug Targeting Strategies Based on Charge Dependent Uptake of Nanoparticles into Cancer Cells. J. Pharm. Pharm. Sci..

[224] Stylianopoulos T., Soteriou K., Fukumura D., Jain R.K. (2013). Cationic Nanoparticles Have Superior Transvascular Flux into Solid Tumors: Insights from a Mathematical Model. Ann. Biomed. Eng..

[225] He Q., Zhang Z., Gao F., Li Y., Shi J. (2011). In Vivo Biodistribution and Urinary Excretion of Mesoporous Silica Nanoparticles: Effects of Particle Size and PEGylation. Small.

[226] Croissant J.G., Fatieiev Y., Khashab N.M. (2017). Degradability and Clearance of Silicon, Organosilica, Silsesquioxane, Silica Mixed Oxide, and Mesoporous Silica Nanoparticles. Adv. Mater..

[227] Trewyn B.G., Nieweg J.A., Zhao Y., Lin V.S.Y. (2008). Biocompatible Mesoporous Silica Nanoparticles with Different Morphologies for Animal Cell Membrane Penetration. Chem. Eng. J..

[228] Wang W., Gaus K., Tilley R.D., Gooding J.J. (2019). The Impact of Nanoparticle Shape on Cellular Internalisation and Transport: What Do the Different Analysis Methods Tell Us?. Mater. Horizons.

[229] Napierska D., Thomassen L.C.J., Rabolli V., Lison D., Gonzalez L., Kirsch-Volders M., Martens J.A., Hoet P.H. (2009). Size-Dependent Cytotoxicity of Monodisperse Silica Nanoparticles in Human Endothelial Cells. Small.

[230] Yu T., Malugin A., Ghandehari H. (2011). Impact of Silica Nanoparticle Design on Cellular Toxicity and Hemolytic Activity. ACS Nano.

[231] Ahmed H., Gomte S.S., Prathyusha A., Agrawal M., Alexander A. (2022). Biomedical Applications of Mesoporous Silica Nanoparticles as a Drug Delivery Carrier. J. Drug Deliv. Sci. Technol..

[232] Guo X., Shi H., Zhong W., Xiao H., Liu X., Yu T., Zhou C. (2020). Tuning Biodegradability and Biocompatibility of Mesoporous Silica Nanoparticles by Doping Strontium. Ceram. Int..

[233] Omar H., Croissant J.G., Alamoudi K., Alsaiari S., Alradwan I., Majrashi M.A., Anjum D.H., Martins P., Laamarti R., Eppinger J. (2017). Biodegradable Magnetic Silica@Iron Oxide Nanovectors with Ultra-Large Mesopores for High Protein Loading, Magnetothermal Release, and Delivery. J. Control. Release Off. J. Control. Release Soc..

[234] Yildirimer L., Thanh N.T.K., Loizidou M., Seifalian A.M. (2011). Toxicological Considerations of Clinically Applicable Nanoparticles. Nano Today.

[235] Couvreur P., Vauthier C. (2006). Nanotechnology: Intelligent Design to Treat Complex Disease. Pharm. Res..

[236] Nelson S.M., Mahmoud T., Beaux M., Shapiro P., McIlroy D.N., Stenkamp D.L. (2010). Toxic and Teratogenic Silica Nanowires in Developing Vertebrate Embryos. Nanomed. Nanotechnol. Biol. Med..

[237] Tang L., Cheng J. (2013). Nonporous Silica Nanoparticles for Nanomedicine Application. Nano Today.

[238] Lu J., Liong M., Li Z., Zink J.I., Tamanoi F. (2010). Biocompatibility, Biodistribution, and Drug-Delivery Efficiency of Mesoporous Silica Nanoparticles for Cancer Therapy in Animals. Small.

